# Characterization of two co-regulated response regulators in *Clostridioides difficile*

**DOI:** 10.1128/jb.00177-25

**Published:** 2025-09-12

**Authors:** Caitlin Lee Williams, Anchal Mehra, Emily K. Harrison, Cynthia G. Thomas, Jack Riley Palmer, D. Kane Cooper, Elizabeth M. Garrett, Rita Tamayo

**Affiliations:** 1 Department of Biology, Virginia Wesleyan University 6044, Virginia Beach, Virginia, USA; 2 Department of Microbiology and Immunology, University of North Carolina at Chapel Hill 318275, Chapel Hill, North Carolina, USA; University of Virginia School of Medicine, Charlottesville, Virginia, USA

**Keywords:** response regulator, pathogenesis, receiver domain, two-component system, *C. difficile*

## Abstract

As an obligate anaerobe, *Clostridioides difficile* grows exclusively in the host intestinal tract, necessitating mechanisms to sense and respond to the gut environment. The atypical signal transduction system encoded by *cmrRST* includes two OmpR-family response regulators, CmrR and CmrT. These regulators control multiple important phenotypes in *C. difficile*, including cell chaining, colony morphology, swimming motility, and biofilm formation. CmrR has a typical receiver domain with key conserved residues for phosphotransfer and conformational change, while CmrT is missing conserved residues and is likely a pseudoreceiver. Here, we used multiple methods to investigate dimer formation, including bacterial two-hybrid systems in both *Escherichia coli* and *C. difficile* and pull-down assays in *E. coli*. We detected CmrR homodimers and found that CmrR recognizes a specific DNA sequence found in multiple places in the genome, including upstream of *cmrRST*. CmrT formed homodimers in multiple assays, and mutation analysis of residues in its unusual active site suggests multiple mutations are needed to maximally reduce CmrT activity. Our data also suggest that CmrR and CmrT can form heterodimers, though the biological relevance of CmrR-CmrT heterodimers remains unclear. Our results suggest that CmrR and CmrT work together in transcriptional responses to stimuli.

**IMPORTANCE:**

Two-component systems are nearly ubiquitous among bacteria and are one of the primary ways that bacteria respond to their environment. Atypical two-component proteins and systems are being identified in diverse bacteria, and studying these proteins helps us to understand the underlying mechanisms of these systems. CmrRST is an unusual two-component system that regulates many important phenotypes in *Clostridioides difficile*. This work begins to untangle the complex regulatory mechanisms by which CmrRST controls gene expression and furthers our understanding of the fundamental biology of *C. difficile*.

## INTRODUCTION

*Clostridioides difficile* is a leading cause of infectious diarrhea and causes approximately 500,000 infections and 30,000 deaths annually in the United States alone (1–3). *C. difficile* is an obligate anaerobe that is transmitted via infectious spores, and the replicating vegetative cells are only found in the gastrointestinal tract of the host. The gut environment is always in flux; therefore, *C. difficile* must constantly adapt for survival and growth. Signal transduction via two-component systems (TCSs) is a common mechanism bacteria use to respond to changes in their environment. The epidemic-associated *C. difficile* strain R20291 encodes more than 40 of these systems, few of which have been studied thus far (4). Canonical TCSs consist of one sensor kinase and one cognate response regulator, and the corresponding genes are typically encoded near one another in the genome and are often co-transcribed. Signal binding alters sensor kinase autophosphorylation activity at a conserved histidine residue. The signal is propagated via transfer of the phosphoryl group to a response regulator. Response regulators have diverse output domains, including DNA-binding domains for transcriptional regulation, ligand-binding domains, and enzymatic domains.

The atypical TCS encoded by the *cmrRST* operon includes one sensor kinase, CmrS, and two response regulators, CmrR and CmrT (5). The *cmrRST* operon was first investigated in connection to two distinct colony morphologies formed by *C. difficile* (5). The rough morphotype is characterized by flatter colonies with irregular, filamentous edges and the smooth morphotype by uniform, circular colonies. The *cmrRST* genes are expressed at a higher level in the rough morphotype than in the smooth. The system was thus named *cmr* for colony morphology regulator. Studies with wild-type rough and smooth variants and mutational analyses further linked increased *cmrRST* expression to several other phenotypes, including increased surface motility, cell elongation and chaining, reduced swimming motility, and reduced biofilm formation. Notably, altering expression levels of *cmrR* or *cmrT* results in distinct phenotypes. Specifically, CmrT, but not CmrR, is required for rough colony formation, but only the *cmrR* deletion mutant has increased biofilm formation (5). Only CmrR autoregulates the *cmrRST* operon (6). These results suggest distinct roles for CmrR and CmrT in gene regulation.

Response regulators are identified by their highly conserved receiver domain. Receiver domain tertiary structure includes five alpha helices surrounding a sheet with five beta strands, with a number of key conserved amino acid residues in the active site and throughout the domain (7, 8). In the active site, an aspartate residue (D) is the site of phosphorylation. In a phosphorylated active site, the phosphoryl group interacts with a T/S, K, and a Mg^2+^ ion coordinated by a pair of conserved aspartate residues (DD). Through these interactions, phosphorylation stabilizes the active conformation, shifting equilibrium toward this state. The active conformation has an output face consisting of the ɑ4 helix, β5 strand, and ɑ5 helix (8). A large aromatic F/Y residue in β5 turns inward in the active conformation, which is important for allowing interactions to occur at the ɑ4-β5-ɑ5 face. The effector domain of most response regulators, including CmrR and CmrT, is an OmpR-family helix-turn-helix DNA-binding domain (9). Transcription factors in this family typically dimerize at the ɑ4-β5-ɑ5 face and recognize a repeated DNA sequence, with one subunit binding to each repeat.

The CmrRST system is unusual in having two response regulators and even more unusual because CmrT has an atypical receiver domain. CmrR possesses the conserved site of phosphorylation in the active site (D52), while CmrT has a glutamate at the corresponding site (E53), placing CmrT in the aspartate-less receiver (ALR) subclass (10). An analysis of all deposited sequences in 2015 found that 4% of receiver domains are ALRs (10). Like CmrT, 26% of substitutions at the conserved aspartate site were to glutamate (10). Glutamate can structurally mimic phosphorylation, and an aspartate to glutamate substitution often results in constitutive activity of typical receiver domains. Replacing D52 in CmrR with a glutamate (D52E) increases its potency when overexpressed (5). Though this single change is not always sufficient for constitutive activity in atypical domains (10), we infer from previous results that CmrT is in fact constitutively active. CmrT overexpression becomes toxic to *C. difficile* at a much lower level of induction than CmrR, and an E53A mutation in CmrT slightly reduces toxicity (5). The minimal effect of the single mutation suggests that additional amino acids are important for the constitutive activity of CmrT.

CmrR and CmrT are linked by their co-transcription in the *cmrRST* operon, regulation of which is impressively complex (6). CmrR and CmrT are also linked in their roles in *C. difficile* biology and perhaps even interact directly. The idea of CmrR/CmrT heterodimers is intriguing and could create an additional layer of complexity. Response regulators that form heterodimers are rare but not unprecedented (11–16). The goal of this work was to investigate the interactions between CmrR and CmrT to further our understanding of this unusual signal transduction system and how it regulates gene expression in the *C. difficile* genome. We assessed protein interactions and DNA binding by CmrR and CmrT to begin to characterize the functions of these regulatory proteins. Our results show that CmrR and CmrT form both homodimers and heterodimers. We identified a binding site sequence for CmrR used for autoactivation of the *cmrRST* operon. We also identified the DNA sequence upstream of additional genes throughout the genome, suggesting that the CmrR regulon includes additional genes. Our work provides a basis for further investigation of the complex roles of CmrR and CmrT in *C. difficile* biology.

## RESULTS

### Homodimerization of CmrR and CmrT

**Fig 1:**
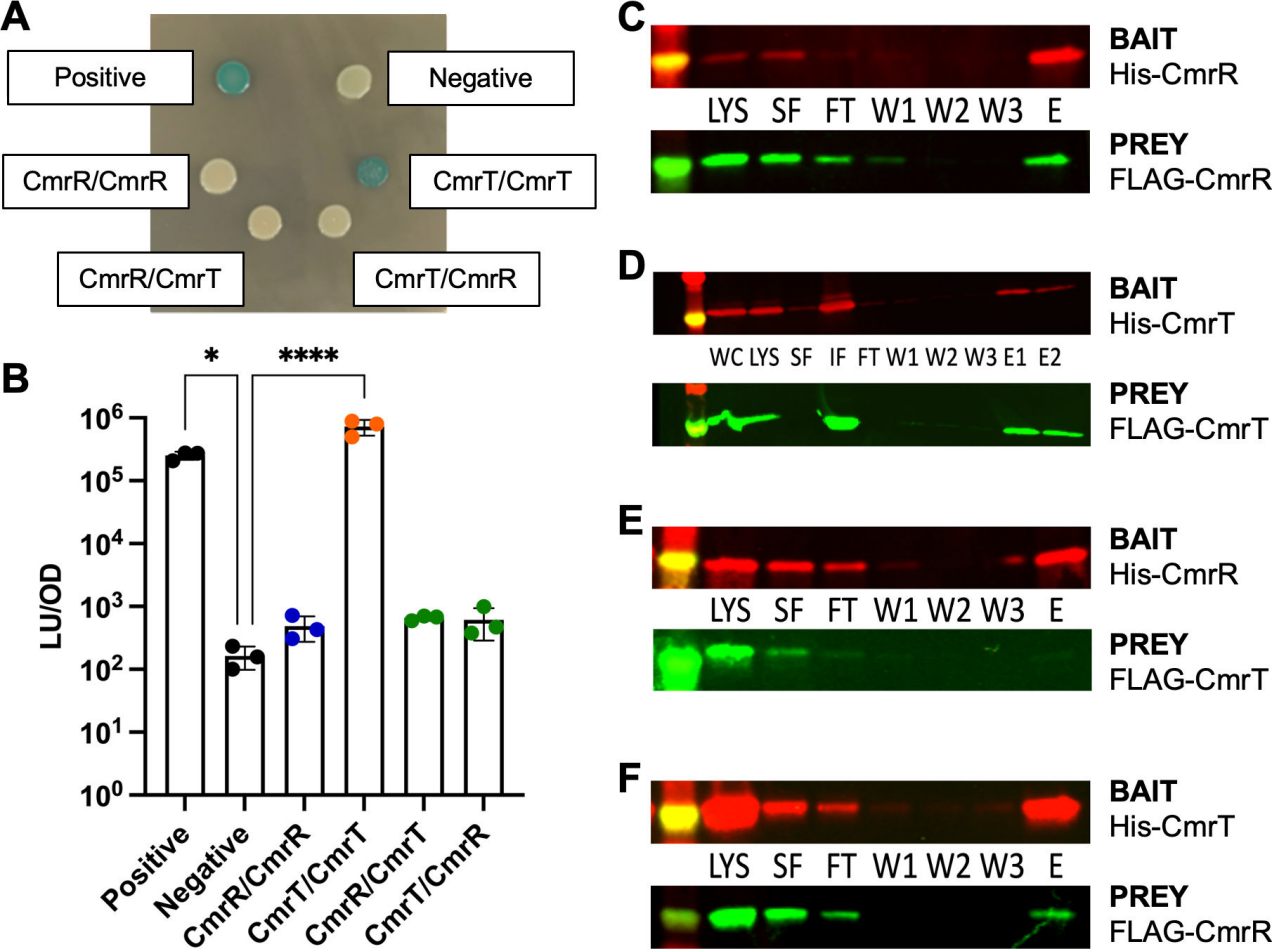
CmrR and CmrT form homodimers and heterodimers. (**A**) A bacterial two-hybrid assay in *E. coli* was used to assess interactions between proteins fused to portions of adenylate cyclase. The positive control expresses two fusion proteins with *zip*, and the negative control strain expresses the halves of adenylate cyclase without translational fusions. Strains were plated on LB agar with IPTG and X-gal. A representative image is shown. (**B**) A bacterial two-hybrid assay in *C. difficile* was used to assess interactions between proteins fused to portions of luciferase. Luciferase signal was measured from strains carrying plasmids encoding the indicated proteins fused to the smbit and lgbit portions of split luciferase, respectively. The positive control strain expresses full-length luciferase enzyme, and the negative control strain expresses split luciferase smbit and lgbit without translational fusions. Data from three biological replicates are plotted with mean and SD. *P* < 0.05, *; *P* < 0.0001, ****; one-way ANOVA compared to negative control with Dunnett’s posttest. (**C-F**) *E. coli* BL21(DE3) was co-transformed with plasmids carrying 6xHis-tagged bait protein and FLAG-tagged prey protein. Strains were grown and induced with IPTG, and proteins were purified by nickel affinity. Samples collected throughout the purification were assessed by Western blot: whole cell, WC; whole cell lysate, LYS; soluble fraction, SF; insoluble fraction, IF; flow through, FT; wash, W; and elution, E. Proteins were separated on two identical SDS-PAGE gels, transferred to nitrocellulose, and probed with anti-His or anti-FLAG antibodies. The band on the far left is the 25 kDa band of the protein ladder. Additional combinations are presented in Fig. S3.

To begin to identify dimerization interactions of CmrR and CmrT, we first chose the *Escherichia coli* bacterial adenylate cyclase two-hybrid (BACTH) system (17). This system uses a split adenylate cyclase enzyme where the protein(s) of interest are translationally fused to each half: T18 and T25. Interactions between the proteins of interest bring the two halves of the enzyme together to assemble a functional enzyme. We assessed enzymatic activity on agar plates with the substrate X-gal, which is cleaved to form a blue product visible in colonies. As expected, spots of the positive control strain appeared blue, while the negative control strain remained white (Fig. 1A; Table S3). To assess the formation of homodimers by CmrR, we translationally fused CmrR to T18 and to T25. We did not observe a color change, indicating no measurable interaction between CmrR monomers (Fig. 1A). We suspected that the lack of homodimerization of CmrR might be due to the lack of the CmrS protein in *E. coli* cells, because we predict that CmrS phosphorylates CmrR to stabilize the active conformation that dimerizes. Therefore, we next utilized a similar bacterial two-hybrid assay developed for *C. difficile* (18). This system utilizes a split luciferase enzyme with each portion of the enzyme translationally fused to a protein of interest. Both fusion proteins are translated from a single transcript controlled by an anhydrotetracycline (ATc)-inducible promoter. Luciferase activity from holoenzyme is detected as output of light from the substrate luciferin. We observed a low level of background luminescence (equivalent to empty wells of the plate) in a strain carrying the negative control plasmid encoding each half of luciferase without a translational fusion (Fig. 1B). High luminescence was observed from the strain carrying the positive control plasmid encoding full-length luciferase. In our hands, the assay had an average 2.25-log_10_ range between the background luminescence of the negative control and the signal from the positive control. To assess the formation of homodimers by CmrR, we translationally fused CmrR to each half of the luciferase enzyme. We did not observe increased luciferase activity above background levels, indicating no measurable interaction between CmrR monomers (Fig. 1B). The apparent lack of dimerization of CmrR could have a number of causes: (i) CmrR requires phosphorylation by CmrS, but this does not occur at a high frequency because CmrS levels are insufficient and/or an activating signal for CmrS is absent in these growth conditions, (ii) two-hybrid tags alter CmrR structure and interrupt dimerization, and/or (3) CmrR does not form homodimers, though this would be surprising given the near universality of this phenomenon in the protein family. The signal that CmrS responds to is unknown, so we opted to increase the relative amount of CmrS in the cell. To do this, we generated rough colony isolates, which have ~10-fold higher transcript abundance of *cmrRST* than the smooth isolates formed by our wild-type isolate (5). We cultured the rough isolates on supplemented brain heart infusion (BHIS) agar, which was previously shown to promote *cmrRST* expression (5). However, luciferase signal was not substantially different in cells grown in these conditions (C. Williams, data not shown). To assess the functionality of the luciferase-tagged versions of CmrR, the fusion proteins were overexpressed in a surface motility assay. In this assay, overexpression of wild-type CmrR increases surface spreading (5). The tagged CmrR protein was compromised in its ability to increase surface motility, suggesting that the fusion proteins retain only partial function compared to the wild-type protein (Fig. S2A).

To assess the formation of homodimers by CmrT, we similarly fused CmrT to T18 and T25 for the *E. coli* bacterial two-hybrid system. When plated on LB agar with isopropyl β-D-1-thiogalactopyranoside (IPTG) and X-gal, interaction from the CmrT/CmrT pair produced blue color comparable to the positive control (Fig. 1A; Table S3). We similarly fused CmrT to each half of the luciferase enzyme for the luciferase-based two-hybrid assay in *C. difficile*. We observed 3.6-log_10_ fold higher activity for the CmrT/CmrT pair compared to the negative control (Fig. 1B). In both two-hybrid assays, the strains expressing only one CmrT fusion protein yielded signal comparable to the negative control, indicating that the interaction was specific to CmrT dimerization and not a result of CmrT interacting with any part of the luciferase enzyme (Fig. S1A, S1E and Table S3). Despite the positive results in the assays for dimerization, the tagged versions of CmrT were also found to be less functional than wild-type CmrT in the surface motility assay (Fig. S2B).

Two-hybrid approaches are limited by the necessity of adding relatively large tags to the proteins of interest, and negative results in a two-hybrid can result from the impact of the tag on protein function rather than a true indication of lack of dimerization. To circumvent this problem, we next assessed dimerization of these proteins using an *E. coli*-based protein pull-down assay with different, smaller epitope tags. We expressed a 6xHis-tagged bait protein with affinity to a nickel resin simultaneously with a FLAG-tagged prey protein. The tags were independently detected via Western blot, allowing us to detect dimers if one subunit had a 6xHis tag and the other subunit had a FLAG tag. Due to the possible low abundance of dimers, especially without CmrS to phosphorylate CmrR, cross-linking was performed at the end of the induction period to stabilize dimers during purification. With this approach, we observed co-purification of FLAG-tagged CmrR with the His-tagged CmrR, suggesting the presence of CmrR homodimers (Fig. 1C). We also observed co-purification of FLAG-tagged CmrT with His-tagged CmrT, though the FLAG-tagged CmrT was mostly insoluble, and the culture volume had to be increased to ensure sufficient soluble prey protein (Fig. 1D). Negative control assays with only bait or only prey demonstrated that the co-purification was not due to interaction between the FLAG-tagged prey protein and the nickel resin (Fig. S3A through D). Cumulatively, these studies suggest that CmrR and CmrT have the capacity to form homodimers.

### Heterodimerization of CmrR and CmrT

Because CmrR and CmrT are encoded in an operon, we were curious about potential interactions between the two proteins, specifically if they form heterodimers. In the *E. coli* and *C. difficile* two-hybrid assays, no signal was detected for heterodimers (Fig. 1A and B). However, as stated above, there are multiple reasons why heterodimerization might not be detected in these assays even though it may occur naturally. In pull-down assays with differentially tagged proteins, interaction between CmrR and CmrT was detected, both when CmrT was pulled down by CmrR and vice versa (Fig. 1E and F). To ensure that this interaction was specific and not due to experimental conditions that encourage dimerization of any two response regulators, we tested for interactions of CmrR and CmrT with another response regulator, CDR20291_2188. Like CmrR and CmrT, this protein has a receiver domain and an OmpR-family DNA-binding domain. Based on a BLASTP search of the proteins encoded in the R20291 genome, CDR20291_2188 is the most similar to CmrR, with 39% identity and 62% similarity across the entire length of the protein. In comparison, CmrT is one of the least similar homologous proteins to CmrR, with 31% similarity and 51% identity to CmrR across only 78% of the amino acid sequence. Of note, *cmrR* and *cmrT* share only 47.9% identity at the DNA level, suggesting they are unlikely to be the result of gene duplication. No interaction between CmrR and CDR20291_2188 nor CmrT and CDR20291_2188 was detected in pull-down assays (Fig. S3E through H). These results bolster our confidence in the detection of CmrR and CmrT heterodimers, though the functional relevance of heterodimers remains unclear.

### Phosphomimetic mutation enhances activity of CmrR

**Fig 2:**
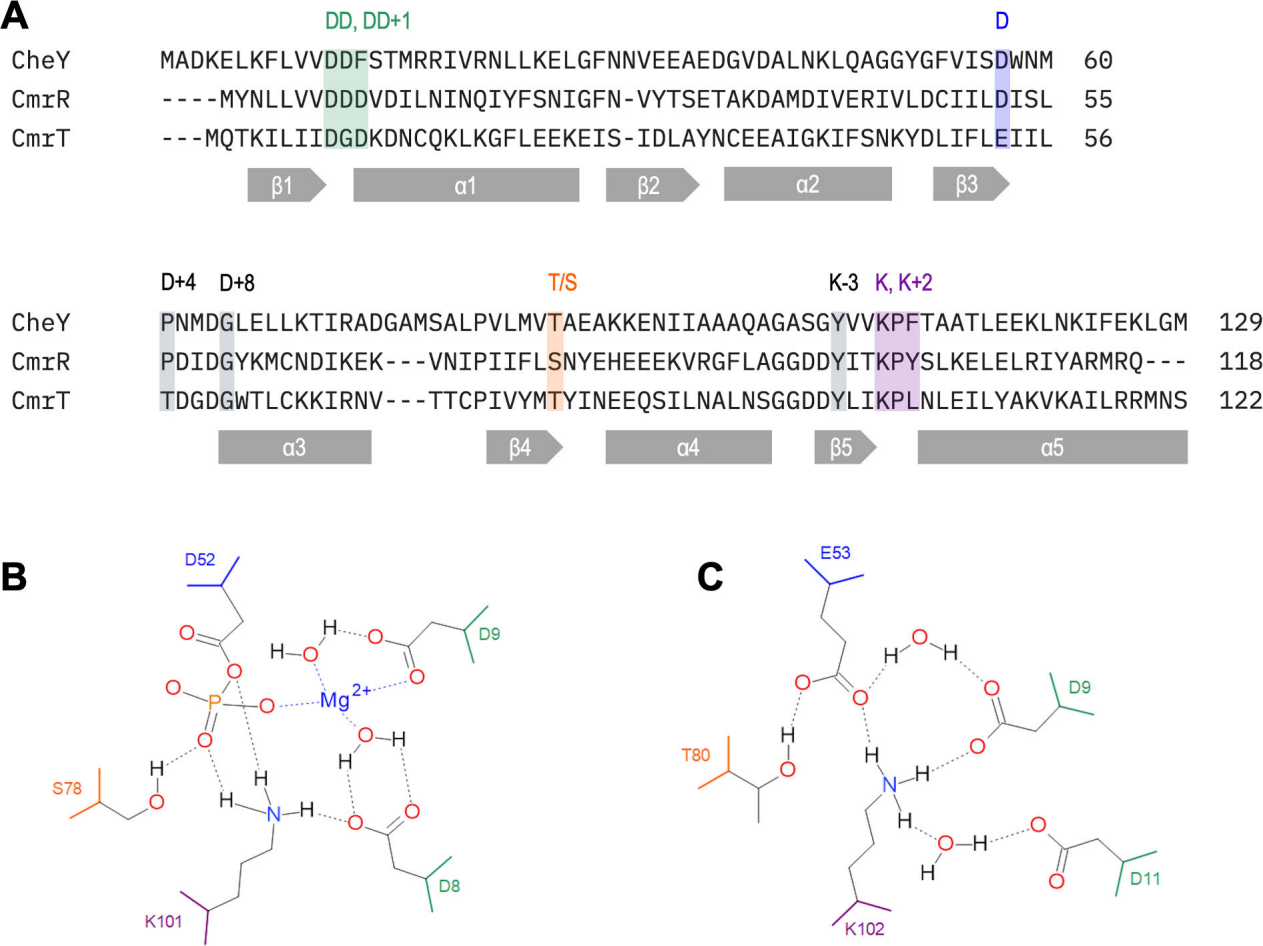
Conserved residues of CmrR and CmrT receiver domains. (**A**) Amino acid sequences of the receiver domains of CmrR (CDR20291_3128, GenBank WP_009891739.1) and CmrT (CDR20291_3126, GenBank WP_009891739.1) were aligned with the canonical CheY (GenBank AAA23577.1) receiver domain using Clustal Omega Multiple Sequence Alignment. Conserved beta sheets and alpha helical structures are represented with gray boxes and arrows, respectively. Important residues in the sequences are highlighted using colors that match the colors in panels B and C with the site names above. (**B and C**) Proposed interactions in the conserved active sites of CmrR (**B**) and CmrT (**C**) were drawn using Biovia Draw (Academic version, 2024). Covalent bonds are represented with solid lines and hydrogen/electrostatic bonds with dashed lines. Bond lengths are not drawn to scale.

**Fig 3:**
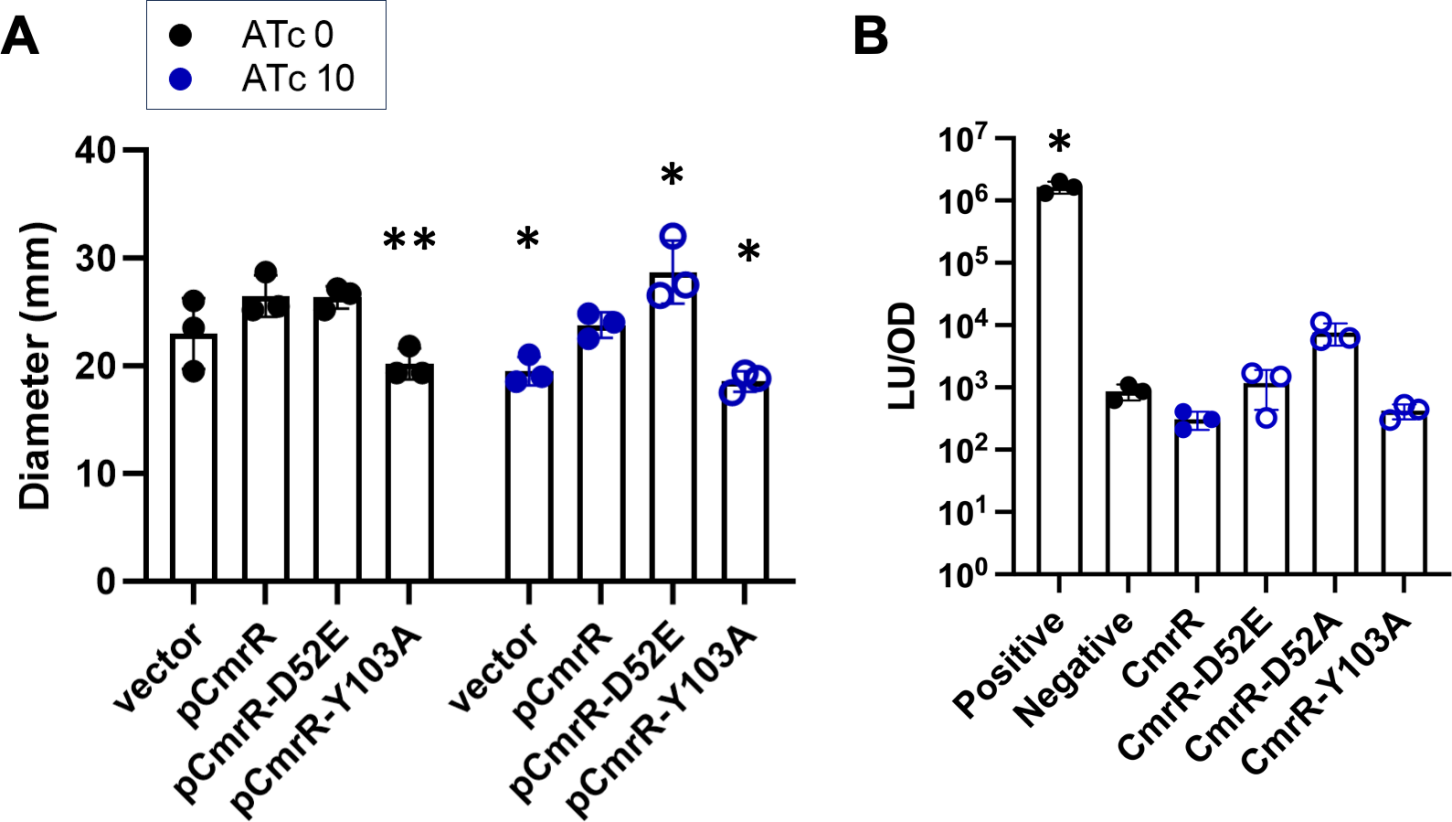
Mutational analysis of CmrR. (**A**) *C. difficile* ∆*cmrR* strains carrying the indicated plasmids were assayed for surface motility on BHIS-1.5% agar with the indicated concentration of ATc. Motility was quantified as the diameter after 7 days of growth. Data from three biological replicates are plotted with mean and SD. *P* < 0.05, *; *P* < 0.01, **; two-way ANOVA compared to pCmrR with the respective ATc concentration using Dunnett’s post-test. (**B**) A bacterial two-hybrid assay in *C. difficile* was used to assess interactions between mutant CmrR proteins. Luciferase signal was measured from strains carrying plasmids with the indicated allele of *cmrR* fused to each half of split luciferase. The positive control strain expresses full-length luciferase enzyme, and the negative control strain expresses split luciferase fragments without a translational fusion. Data from three biological replicates are plotted with mean and SD. *P* < 0.05, *; Brown-Forsythe and Welch ANOVA compared to CmrR with Dunnett’s T3 post-test.

The receiver domain of CmrR possesses all the key conserved residues of a standard receiver domain based on an alignment of the CmrR amino acid sequence with the prototypical CheY receiver domain (Fig. 2A) (8). In the active site, CmrR has the conserved phosphoryl-receiving aspartate residue (D52), DD motif (D8, D9), T/S (S78), and K (K101) (Fig. 2B). CmrR also maintains the conserved proline in the D+4 position, glycine at D+8, and F/Y at K-3. Because CmrR has all the expected conserved residues in its receiver domain, we anticipate that CmrR forms dimers when in the active conformation. Because phosphorylation stabilizes the active conformation that forms the dimerization face, the lack of a signal from CmrS could contribute to the difficulty in detecting CmrR homodimers. Therefore, we made a “phosphomimic” mutation in CmrR, D52E, that we expected to artificially activate the protein by encouraging the conformational change associated with phosphorylation. Previous results from the lab showed that CmrR-D52E is more potent than wild-type CmrR in producing *cmr*-associated phenotypic changes in *C. difficile* (5). We again observed this phenomenon, where CmrR-D52E induced 20% greater surface motility than wild-type CmrR in a *cmrR* mutant strain (Fig. 3A). Similar patterns among strains were observed with and without the addition of 10 ng/mL ATc for induction, presumably due to the known leakiness of the ATc-inducible promoter. Unexpectedly, CmrR-D52E did not produce higher activity in either bacterial two-hybrid assay, suggesting no increase in dimerization or insufficient sensitivity of the assay (Fig. 3B; Fig. S1D and Table S3). This contrast may again point to interference in protein function by the large translational fusions used for the two-hybrid assays (Fig. S2), as untagged versions of CmrR-D52E appear to be functional (Fig. 3A).

Because CmrR-D52E did not behave as expected in the two-hybrid assays, we investigated another possible substitution that might promote dimerization to a detectable level. An alanine substitution in the K+2 position of the prototypical CheY receiver domain was shown to significantly increase rates of autophosphorylation and the proportion of the population in the active conformation (19). The K+2 position in CmrR corresponds to residue Y103, suggesting that a Y103A mutation could similarly activate CmrR. However, with expression of CmrR-Y103A, we did not observe activity above background levels in the *C. difficile* two-hybrid assay nor enhanced surface motility (Fig. 3A and B). Taken together, these results suggest the phosphomimic D52E mutation enhances CmrR activity, while the Y103A mutation has no effect.

### Multiple mutations are required to inactivate CmrT

Although CmrT maintains many key residues conserved in receiver domains, it is an atypical ALR that lacks the conserved phosphoryl-receiving aspartate in the active site. Instead of aspartate, CmrT encodes a phosphomimetic glutamate residue at this position, E53. Another deviation is at the DD motif, which is often followed by another D at DD+1. In CmrT, this is an unusual DGD motif (D9, G10, D11). In standard receiver domains, these residues are important for coordinating Mg^2+^ and interacting with the phosphorylated aspartate (as in CmrR, see Fig. 2B) (7). CmrT retains the conserved T/S (T80) and K (K102) residues of the active site (Fig. 2A). Among the ALRs, this pattern of substitutions is common, with the phosphorylation site D and the second D of the DD pair being the most likely to be altered (10). The crystal structure of the CmrT receiver domain, amino acids AA 1-128, was solved and is available in Protein Data Bank (structure 2QZJ) (20) and suggests the residues of the would-be active site interact directly, without the need for a Mg^2+^ or phosphorylation (Fig. 2C). Hydrogen bonding can be observed between D9, D11, E53, T80, and K102 (Fig. 2C). These interactions mimic those of a standard receiver domain in the active conformation (10). The CmrT crystal structure was included in a previous analysis of ALR structures which found that hydrogen bonding among the residues of would-be active sites was common among the analyzed ALR structures (10). That study noted the interactions among D9, E53, and K102. But, in part because of the unusual DGD motif of CmrT, they did not note the interactions with D11 and T80. In our model, these additional interactions further stabilize the would-be active site of CmrT (Fig. 2C). We hypothesized that the hydrogen bonding among residues in the would-be active site of CmrT stabilizes the protein in the active conformation, which facilitates dimerization and DNA binding.

**Fig 4:**
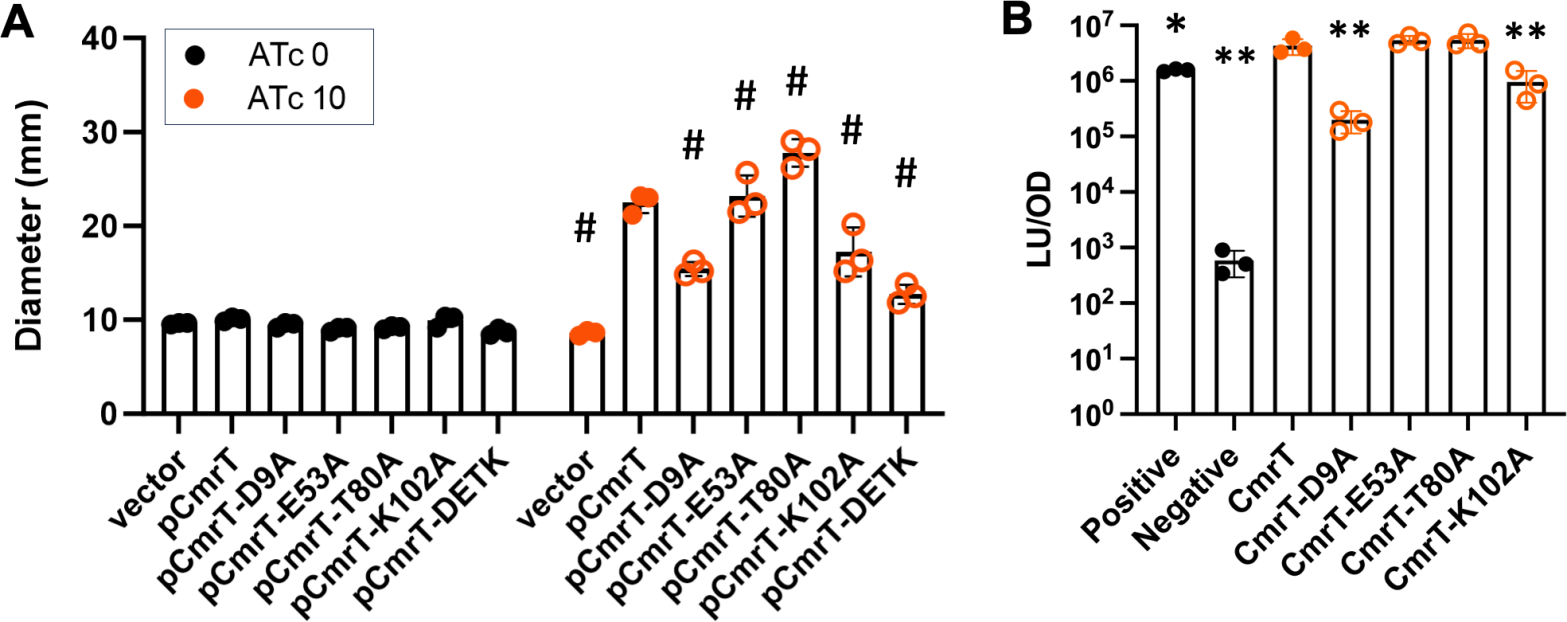
Mutational Analysis of CmrT. (**A**) *C. difficile* ∆*cmrR*∆*cmrT* strains carrying overexpression plasmids with the indicated *cmrT* alleles were assayed for surface motility on BHIS-1.5% agar with the indicated concentration of ATc. Motility was quantified as the diameter after 7 days of growth. Data from three biological replicates are plotted with mean and SD. *P* < 0.0001, #; two-way ANOVA compared to pCmrT with Dunnett’s posttest. (**B**) The *C. difficile* two-hybrid assay was used to assess interactions between CmrT variants. Luciferase signal was measured from strains carrying plasmids encoding the indicated allele of *cmrT* fused to each half of the split luciferase. The positive control strain expresses full-length luciferase enzyme, and the negative control strain expresses split luciferase fragments without translational fusions. Data from three biological replicates are plotted with mean and SD. *P* < 0.05, *; *P* < 0.01, **; one-way ANOVA compared to CmrT with Dunnett’s post-test.

To investigate the importance of active site residues of CmrT, we conducted a mutational analysis and assayed for effects on protein-protein interactions and on phenotypic changes resulting from regulation of gene expression. We made alanine substitutions at the would-be active site residues D9, E53, T80, and K102 alone and in combination. We measured the effect of these mutations on CmrT function *in vivo* by assaying surface motility, which requires CmrT. We overexpressed the *cmrT* alleles in a Δ*cmrR*Δ*cmrT* mutant, which is incapable of surface motility but can be complemented with *cmrT* expressed *in trans* (Fig. 4A). Our lab previously showed that overexpression of the *cmrT*-E53A allele results in an increase in surface motility similar to that of wild-type *cmrT*, suggesting this single substitution is insufficient to significantly alter CmrT function (5). In contrast, CmrT with a D9A or K102A mutation was deficient in restoring surface motility, with an average decrease in motility of 31% and 23% relative to wild type (Fig. 4A). We evaluated homodimer formation by the CmrT mutants using the *C. difficile*-based bacterial two-hybrid assay. Consistent with the results of the surface motility assay, the E53A and T80A mutations had no substantial impact, while the D9A and K102A mutations resulted in reduced interaction (−1.27-log_10_ fold change with D9A and −0.64-log_10_ fold change with K102A, Fig. 4B). We additionally generated a quadruple mutant of CmrT with D9A, E53A, T80A, and K102A substitutions (referred to as DETK) for the surface motility assay. The combined mutations resulted in the most severe surface motility defect with a 43% loss in motility compared to wild-type CmrT. All of the mutations caused statistically significant differences in motility when compared to wild-type CmrT (Fig. 4A). In an alternative statistical analysis to compare the mutations to one another, we used a one-way analysis of variance (ANOVA) and Tukey’s post-test to compare the data for the induced condition, and we found that the quadruple mutant had significantly lower surface motility than the T80A and K102A mutants (*P* < 0.0001, *P* = 0.038, respectively). These results support our hypothesis that these active site residues are important for CmrT function and our inference that the ability to form dimers is crucial for CmrT to function as a transcription factor.

### CmrR binds upstream of *cmrRST*

**Fig 5:**
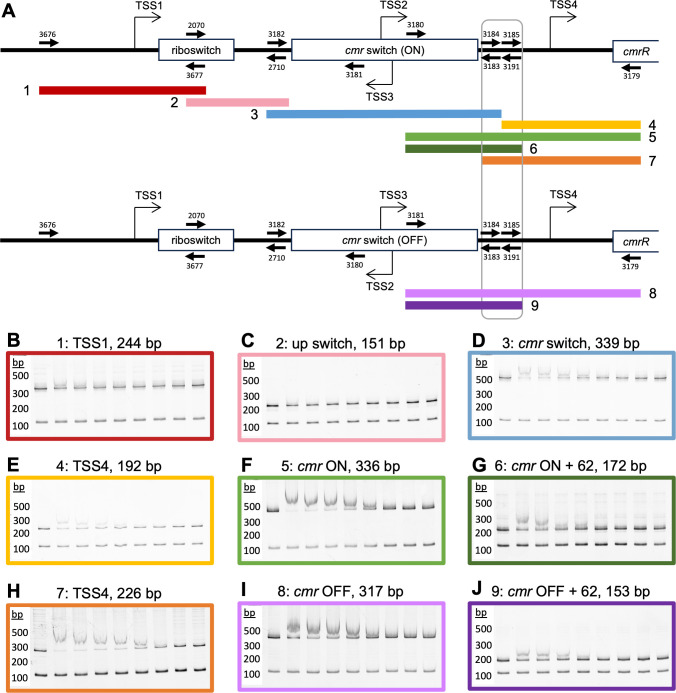
Identification of a CmrR Binding Site. (**A**) A diagram of the regulatory region upstream of *cmrRST,* drawn to scale, with four transcriptional start sites depicted by arrows (TSS1-4) and the c-di-GMP riboswitch and *cmr* switch indicated. The locations of the DNA probes used for EMSAs are indicated by colored lines and labeled with the probe number. PCR primers are depicted as arrows labeled with the primer number. The location of the identified binding site upstream of TSS4 is boxed in gray. (**B–J**) Purified 6xHis-tagged CmrR was tested for the ability to bind regions upstream of *cmrRST* using EMSAs. Dilutions of CmrR were incubated with 15 nM of the indicated probe and a 100 bp *rpoC* control probe. The approximate concentration of CmrR in each reaction from left to right was 0, 1.5, 1.3, 1.1, 0.9, 0.7, 0.5, 0.3, and 0.04 µM. Bands were imaged with GelRed. A representative image of two independent experiments is shown. DNA ladder band sizes are indicated.

Previous results from the lab showed that CmrR positively autoregulates expression of the *cmrRST* operon (6). Four transcriptional start sites were identified upstream of the operon: TSS1 is furthest upstream and followed by a c-di-GMP riboswitch, TSS2 is within the *cmr* switch and oriented toward *cmrRST* when the switch is in the ON orientation, TSS3 is also within the *cmr* switch and oriented toward the operon when the switch is in the OFF orientation, and TSS4 is between the *cmr* switch and the *cmrR* coding sequence (Fig. 5A). Of note, the transcriptional activity from TSS3 was extremely low and is not considered a bona fide TSS (6). Reporter assay experiments testing various portions of the *cmrRST* promoter showed that *cmrR* overexpression only increased reporter activity when the promoter region included TSS4, suggesting that the autoregulation by CmrR occurs at TSS4 (6). To identify the CmrR binding site for *cmrRST*, we used electrophoretic mobility shift assays (EMSA) to test for binding of purified CmrR to DNA fragments corresponding to portions of the region upstream of *cmrRST* (Fig. 5A). We observed direct binding of CmrR to DNA fragments that contain a 62 bp sequence found 40–102 bases upstream of TSS4, boxed in gray in Fig. 5A. Binding of CmrR to all probes containing this region suggests the CmrR binding site lies within the 62 bp sequence (probes 5–9; Fig. 5F through J). Use of truncated probes missing half of the 62 bp region resulted in weaker binding, suggesting the truncation divided the CmrR binding site (e.g., probe 4 vs. probe 7, Fig. 5E and H). Binding was not affected by the orientation of the *cmr* switch (e.g., probe 5 vs probes 8, Fig. 5F and I ), indicating the CmrR does not bind to the invertible sequence of the switch. We also did not observe binding to fragments containing sequences found further upstream (Fig. 5B and C). Taken together, these results suggest that CmrR binds within the region 40–102 bases upstream of TSS4.

### Identification of the CmrR consensus sequence

**Fig 6:**
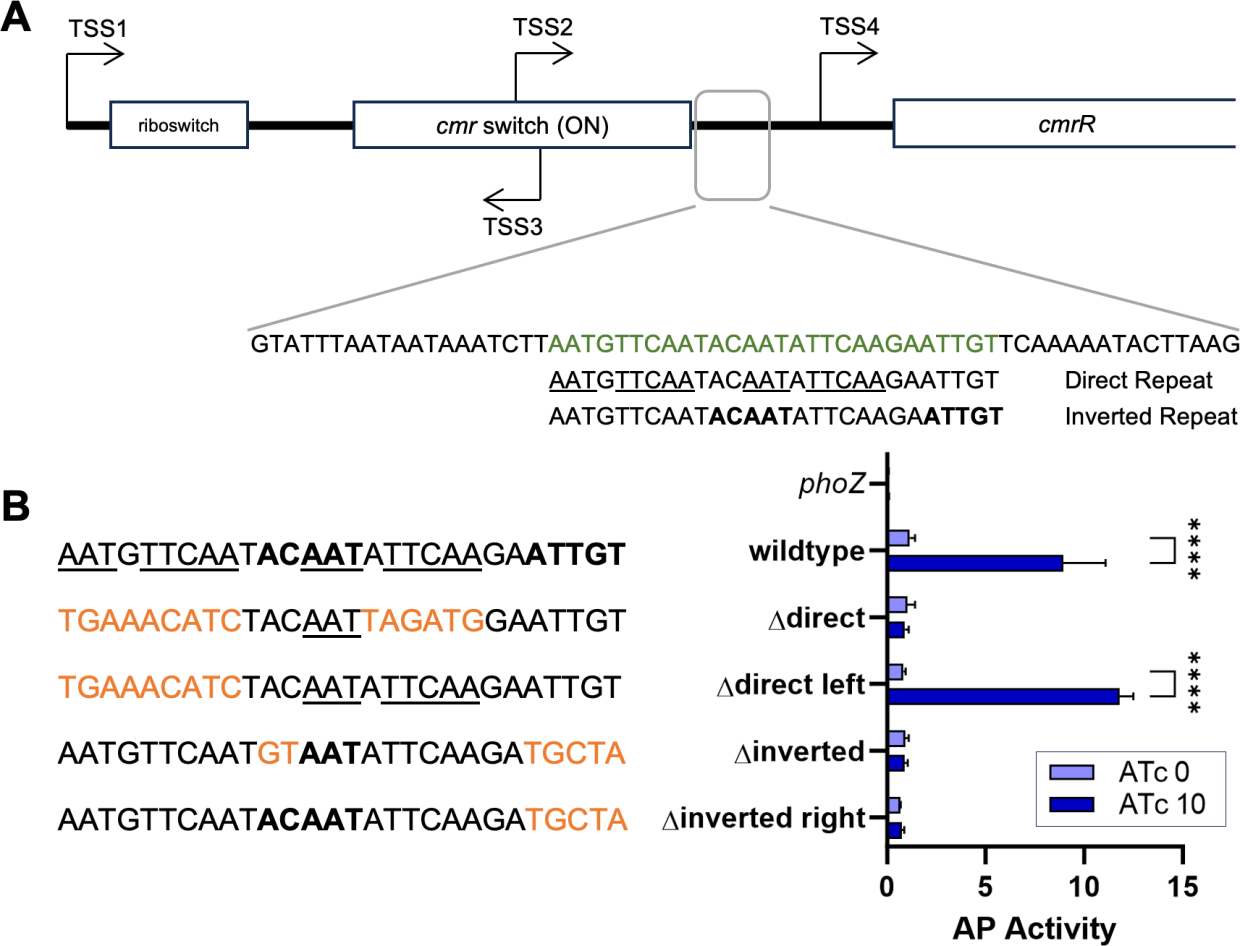
Identification of a CmrR binding site sequence. (**A**) Diagram of the regulatory region upstream of *cmrRST*. The 62 bp region containing the identified binding site upstream of TSS4 is boxed in gray. The sequence of the 62 bp region is below, with the sequence containing the binding sites indicated in green. The direct and inverted repeat sequences of interest are indicated with underlined or bolded font, respectively. (**B**) Alkaline phosphatase (AP) assays were used to measure promoter activity from wild-type and mutated versions of the *cmrRST* promoter region containing TSS4. Promoter sequences are shown with mutated nucleotides in orange, and the unaltered bases of the repeating sequences still underlined or bolded. Strains of *C. difficile* with ATc-inducible P*_tet_::cmrR* at an ectopic chromosomal site were grown with plasmid-borne reporter fusions containing the indicated sequence. AP reporter activity was measured after growth with or without 10 ng/mL ATc to induce *cmrR* expression. Shown are means and SD from three independent experiments. ****, *P* < 0.0001; two-way ANOVA with Sidak’s post-test.

Response regulators with OmpR-family helix-turn-helix DNA-binding domains typically recognize repeated sequences with each subunit of the dimer interacting with one repeat of the binding site (21). The repeated sequences can be either direct or inverted, and they are typically six to eight nucleotides long with an intervening ~5 bp spacer, for a total length of 15–20 bases. We manually searched the 62 bp region that was bound by CmrR in the EMSAs for repeat sequences that fit these criteria. We identified a region with two potential binding sites, one with direct repeats and the other inverted (Fig. 6A, underlined and bolded sequences). Notably, both sites were disrupted in the probes that showed weak binding (e.g., probe 4 vs. probe 7; Fig. 5E and H). To determine if either of these repeat sequences are the CmrR binding site, we mutated several bases and used a *phoZ* transcriptional reporter to measure promoter activity in response to overexpression of *cmrR*. To maximize the potential effects of the mutations to the nucleotide sequence, the initial set of mutated promoters had changes to all the nucleotides of the direct repeat sequence that were not part of the inverted repeat (∆direct) and vice versa (∆inverted) (Fig. 6B, orange bases). Neither the ∆direct nor ∆inverted mutated promoters showed increased activity when *cmrR* was overexpressed (Fig. 6B), suggesting that the mutations to the promoter were too drastic, and both altered the CmrR binding site enough to lose binding. We therefore constructed new reporters with changes only to nucleotides in the non-overlapping repeats. Specifically, we changed the left half of the direct repeat (∆direct-left) and the right half of the inverted repeat (∆inverted-right) so that no changes were made in the overlapping region. The ∆direct-left promoter maintained responsiveness to CmrR, indicating that the direct repeat sequence is likely not the CmrR binding site. However, CmrR failed to activate the ∆inverted-right mutated promoter, indicating that the CmrR binding site was eliminated with this mutation and CmrR recognizes the inverted repeat sequence, **ACAAT**ATTCAAGA**ATTGT**.

### Identification of additional CmrR binding sites in the genome

**Fig 7:**
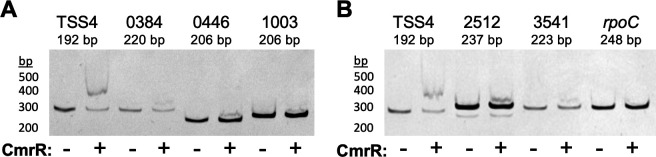
CmrR binds to other sites in the chromosome. Purified 6xHis-tagged CmrR was tested for its ability to bind DNA probes containing the putative CmrR consensus binding sequence using EMSA. The indicated DNA probes were incubated with 0 or 1.2 µM CmrR. Probes were tested simultaneously and analyzed on two gels in parallel. A probe containing TSS4 (probe 7 in Fig. 5) was included as a positive control (**A, B**) and a *rpoC* probe was included as a negative control (**B**). Representative images from two independent experiments are shown. DNA ladder band sizes are indicated.

With the identified binding sequence, we used the MEME Suite’s FIMO program to search the R20291 genome for the sequence ACAATNNNNNNNNATTGT. Twelve exact matches were identified, including the site upstream of *cmrRST*, and we chose five sites in putative promoter regions for further investigation because of their location upstream of genes (Table 1). The genes downstream of the identified sites have various predicted functions, including a regulator of arginine utilization (CDR20291_0384), another TCS (CDR20291_0446), a homoserine dehydrogenase (CDR20291_1003), a putative exported protein (CDR20291_2512), and 50s ribosomal protein L34 (CDR20291_3541). We tested each of the sites for binding by CmrR using EMSAs. For these 5 probes, we observed a minor band running at a higher molecular weight only when incubated with CmrR, suggesting they are bound by CmrR. However, binding may be weak, as the bands shifted less with these candidates than with the *cmrRST* TSS4 probe (Fig. 7A and B). These results suggest that CmrR has the potential to directly regulate additional genes in the R20291 genome.

**TABLE 1:** Identified consensus sequence sites in the *C. difficile* R20291 chromosome

Identified sequence	Location in FN545816	Downstream gene	Distance from start codon (bp)
Consensus: ACAATNNNNNNNNATTGT			
ACAATTAATTAAGATTGT	457,664	CDR20291_0381/CDR20291_0382	NA^*a*^ – between two stop codons
ACAATTTCTATAGATTGT	460,454	CDR20291_0384 (*rocR*)	377
ACAATAAGGAGGAATTGT	537,144	CDR20291_0446	23
ACAATTGGAAGAAATTGT	1,219,792	CDR20291_1003	259
ACAATAATTGATAATTGT	2,947,964	CDR20291_2512	75
ACAATAAATCTAAATTGT	3,057,494	CDR20291_2605	NA – within ORF
ACAATCTCAAGAAATTGT	3,300,497	CDR20291_2787 (*ntpD*)	NA – within ORF
ACAATGAACTGCTATTGT	3,625,502	CDR20291_3037	NA – within ORF
ACAATATTCAAGAATTGT	3,736,114	CDR20291_3128 (*cmrR*)	146
ACAATCATCAACTATTGT	3,752,955	CDR20291_3143 (*pflD*)	NA – within ORF
ACAATTACCAGTCATTGT	3,792,364	CDR20291_3179 (*fdhF*)	NA – within ORF
ACAATATTAACTAATTGT	4,190,950	CDR20291_3541 (*rpmH*)	269

^*a*^ NA, not applicable.

## DISCUSSION

The CmrRST system of *C. difficile* is an example of an unusual signal transduction system with three components: one histidine kinase and two response regulators. The system modulates many phenotypes in *C. difficile*, including colony morphology, cell chaining, swimming and surface motility, and biofilm formation (5). Another unusual feature is that CmrT has an atypical ALR domain. In this study, we investigated the protein-protein interactions of CmrR and CmrT and identified DNA-binding sites of CmrR to begin to understand how this unusual signal transduction system mediates gene regulation and control of multiple important phenotypes in *C. difficile*.

We found that both CmrR and CmrT are capable of forming homodimers using multiple approaches. Although we were only able to detect homodimers of CmrR by co-purification, lack of homodimer formation in the bacterial two-hybrid assays can arise from numerous reasons that make negative results difficult to interpret, such as steric hindrance from the reporter fusions or insufficient activating signal from CmrS. Evidence of CmrT homodimerization was observed in all assays, even in heterologous systems without CmrS, aligning with our inference that its activity is constitutive and not dependent on phosphorylation. The crystal structure of CmrT shows interactions among multiple residues in the would-be active site to stabilize the active conformation and the ɑ4-β5-ɑ5 dimerization face. In our mutational analysis of CmrT, single D9A and K102A mutations in the active site reduced dimerization and function of CmrT, but the quadruple mutation had the greatest impact. These results are consistent with the predicted stability of the CmrT active site, though we cannot rule out unintended effects of the quadruple mutation on overall protein folding and stability. CmrR and CmrT appear to form heterodimers in the pull-down assays, although what this observation means functionally remains unclear.

Mutations in the conserved active site residues DD, D, T/S, and K are common among pseudoreceivers, which were defined in a recent study as receivers missing any of those five key residues for phosphorylation chemistry (22). A handful of atypical and/or ALRs have been previously characterized, some of which have been shown not to require phosphorylation for dimerization and/or activity (10, 23). Many of these unusual response regulators have structural deviations from the conserved receiver domain structure that alter their mode of action (10, 24–28). By comparison, the receiver domain of CmrT has few deviations with only minimal changes of DD to DGD and D to E. A few of the previously characterized atypical response regulators also have minimal substitutions of key conserved residues in the receiver domain (29–32). Of those characterized, the one most akin to CmrT may be AtvR of *Pseudomonas aeruginosa*, a NarL-family response regulator. The receiver domain of AtvR is only missing one of the conserved DD residues to coordinate Mg^2+^ and has the same D to E substitution at the phosphorylation site. AtvR maintains the other key residues, including T/S, K, Y at K-3, P at D+4, and G at D+8. CmrT also maintains these conserved residues except for the P at D+4. Due to the phosphomimetic nature of the D to E substitution at the phosphorylation site, AtvR was also inferred to be constitutively in the active conformation without the need for phosphorylation or involvement of a histidine kinase (29). The inferred constitutive activity of AtvR has not yet been investigated. Of note, a few other response regulators were characterized in *Streptomyces* species that have predicted or demonstrated constitutive activity, despite minimal deviations in the receiver domain: RamR, WhiI, and BldM (30–32). While the mechanism of constitutive activity for RamR is unlike that of CmrT (an L substitution for T/Y at K-3), WhiI and BldM might have constitutive activity due to active site substitutions stabilizing the active conformation. Like we observed with CmrT, single and double mutations were insufficient to abolish activity of WhiI and BldM (31, 32).

If AtvR, RamR, WhiI, BldM, and CmrT are indeed constitutively active, then they act more like a stand-alone transcription factor than a traditional response regulator that is part of a signal transduction pathway. Thus, regulation of their activity must occur via mechanism(s) other than the characteristic phosphorylation-dependent conformation change of standard receiver domains. For AtvR, expression is induced by anoxic conditions, and it is presumed to be active whenever it is expressed (29). Interestingly, WhiI and BldM form heterodimers, which regulates their activity (discussed below). We speculate that the activity of CmrT could be regulated in two non-mutually exclusive ways: (i) complex transcriptional control of *cmrT* expression and (ii) binding to CmrR in heterodimers. These mechanisms of regulation would allow CmrT to maintain relatively normal receiver domain structure while losing the typical signal cascade feature of a TCS.

Transcriptional control of the *cmrRST* operon is impressively complex, with three distinct mechanisms of regulation: a c-di-GMP riboswitch that positively regulates transcription in response to ligand binding, an invertible element whose orientation modulates *cmrRST* expression, and autoactivation via a CmrR-dependent promoter (5, 6, 33–35). The entire regulatory region is highly conserved among *C. difficile* strains (6), which suggests that these mechanisms are important for regulating expression of *cmrRST* and the genes regulated by the system. We found that CmrR binds upstream of TSS4, to an inverted repeat sequence ACAATNNNNNNNNATTGT.

Formation of CmrR/CmrT heterodimers could post-transcriptionally regulate activity of CmrR and CmrT in the cell. Formation of heterodimers by response regulators is uncommon, but not unprecedented. Systematic testing of combinations of response regulators in *E. coli* demonstrated numerous hetero-pair interactions, nearly all with lower affinity than their respective homodimers (13). A few heterodimer pairs have been experimentally confirmed: NarL/DevR in *Mycobacterium tuberculosis*, DevR/PhoP in *M. tuberculosis,* EssR/CenR in *Brucella ovis*, BldM/WhiI in *Streptomyces*, and RcsB forms heterodimers with many response regulators in *E. coli* (11, 12, 14–16). Like CmrT, BldM and WhiI also have atypical receiver domains, and as seen with CmrR and CmrT, BldM can form a homodimer or a heterodimer with WhiI (11). Of note, none of the genes encoding these heterodimer partners are co-transcribed like *cmrR* and *cmrT*, so the additional mechanism of transcriptional control of *cmrT* by CmrR is an unusual added layer of interaction. BldM homodimers bind a typical inverted repeat sequence, and the heterodimer binding site has one repeat of the BldM sequence and a different sequence for the subunit of WhiI (11). The CmrR/CmrT heterodimer may similarly bind a single repeat of the CmrR sequence alongside a single repeat of the sequence recognized by CmrT. Identification of the binding sequence for CmrT is necessary for predicting putative heterodimer binding sites bioinformatically. Transcriptional studies will be used to define the CmrR and CmrT regulons, which will aid in the search for the CmrT binding site sequence.

**Fig 8:**
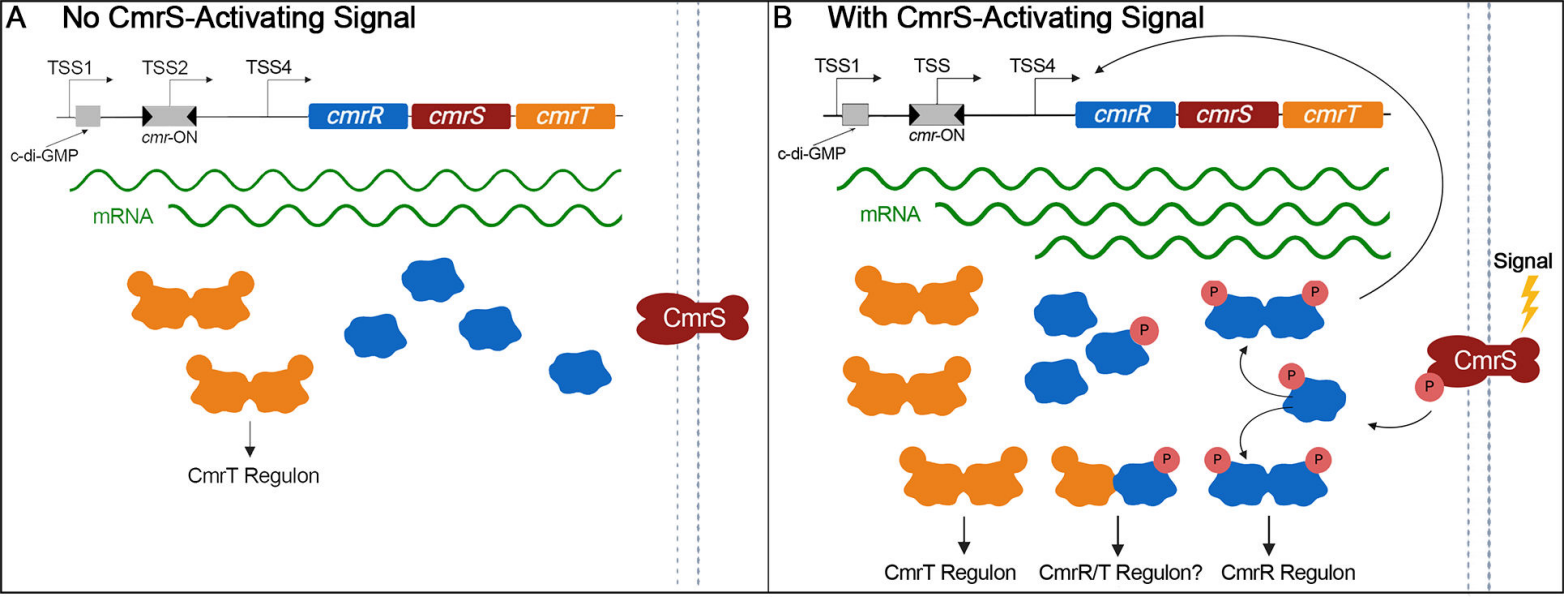
Model of CmrRST signaling and regulation. Two of multiple possible scenarios based on the level of *cmrRST* transcription and the presence of a CmrS activating signal are presented. (**A**) In the absence of an extracellular signal for CmrS, *cmrRST* expression is determined by intracellular c-di-GMP level and *cmr*-ON/OFF state. Commensurate production of constitutively active CmrT leads to regulation of the CmrT regulon. CmrR and CmrS are also produced, but the lack of a stimulus maintains this system in an inactive state. (**B**) Upon sensing a signal, CmrS autophosphorylates and activates CmrR via phosphotransfer. Phosphorylated CmrR dimerizes and autoactivates expression of *cmrRST*, amplifying the response. CmrR dimers may control additional genes. We speculate that CmrT and phosphorylated CmrR may form heterodimers and regulate a distinct regulon.

Based on our results and our understanding of similar response regulators, we propose a model in which CmrR/CmrR homodimers, CmrT/CmrT homodimers, and CmrR/CmrT heterodimers regulate three distinct gene sets in different environmental conditions (Fig. 8). The existence of multiple mechanisms controlling *cmrRST* transcription provides *C. difficile* with the ability to express the operon under a variety of conditions, helping ensure that the system is present and able to respond to a CmrS stimulus. In the absence of a CmrS-activating signal, the amount of CmrRST in the cell would depend on intracellular c-di-GMP levels and the ON/OFF status of the *cmr* switch (Fig. 8A). CmrR would remain primarily monomeric, while any CmrT present would dimerize and regulate target genes due to its constitutively active state. In the presence of an activating signal, CmrS-mediated phosphorylation of CmrR would promote formation of CmrR homodimers (Fig. 8B). The resulting autoactivation of *cmrRST* transcription would increase the abundance of CmrRST and potentially amplify the response to the stimulus, as well as increase the amount of CmrT and modulation of its regulon. CmrR may directly regulate additional genes, though this has not been demonstrated. Based on our results, we postulate that phosphorylation of CmrR promotes heterodimerization with CmrT and that heterodimers control expression of a third set of genes (Fig. 8B).

Additional work is needed to test our model and disentangle the web of interactions between CmrS, CmrR, and CmrT. A more complete understanding of the CmrRST signaling system and the distinct roles of CmrR and CmrT in *C. difficile* physiology and virulence will expand our knowledge of atypical two-component signaling pathways and fundamental mechanisms of this important pathogen, which is a key step toward development of improved therapeutic options.

## MATERIALS AND METHODS

### Strains and culture conditions

Strains and plasmids used in this study are described in Table S1. *E. coli* strains were routinely cultured in Luria Bertani (LB) medium (Fisher BP1426) at 37°C with aeration, with antibiotics when appropriate. *C. difficile* strains were cultured in a Coy anaerobic chamber with an atmosphere of 5% carbon dioxide, 10% hydrogen, and 85% nitrogen. *C. difficile* was grown in pre-reduced supplemented (BHIS) medium (37 g/L Bacto brain heart infusion, 5 g/L yeast extract) with 0.1% L-cysteine or Tryptone Yeast (TY) medium (30 g/L Bacto tryptone, 20 g/L yeast extract, 1 g/L thioglycolate) with antibiotics when appropriate. All *C. difficile* cultures, in liquid and solid media, were incubated statically at 37°C unless otherwise specified. Antibiotics were used at the following concentrations: 10–20 μg/mL chloramphenicol (Cm10 or Cm20), 10 µg/mL thiamphenicol (Tm10), 50–100 μg/mL kanamycin (Kan50 or Kan100), and 100 µg/mL ampicillin (Amp100). Details on cloning and mutant generation are provided in Supplemental Materials.

### *E. coli* bacterial two-hybrid assays

BTH101 strains were streaked from freezer stocks on LB-Amp100-Kan50 agar. Colonies were suspended in 2 mL LB broth to an OD_600_ of 0.2, and 5 µL of the suspension was spotted on LB agar plates with Amp100, Kan50, 0.5 mM IPTG, and 40 µg/mL 5-bromo-4-chloro-3-indolyl-β-D-galactopyranoside (X-gal). Plates were incubated at 30°C for 48 hours before imaging via standard photography.

### *C. difficile* bacterial two-hybrid assays

Frozen stocks were streaked on BHIS-Tm10 agar plates, and single colonies were used to inoculate overnight cultures in TY-Tm10 broth. Overnight cultures were diluted in BHIS-Tm10 and grown to exponential phase. Expression of fusion proteins was induced by the addition of 200 ng/mL ATc for exactly 1 hour, at which time cultures were removed from the anaerobic chamber. The final OD_600_ was measured for normalization, and 100 µL of room temperature culture was added to 20 µL of room temperature Nano-Glo Luciferase Assay Reagent (Nano-Glo Luciferase Assay System, Promega) in a white 96-well plate (Grenier LUMITRAC, Bio-One). After 13 minutes of incubation, luminescence was measured in a BioTek Synergy H1 Hybrid Reader microplate reader with 0.1 s exposure and high sensitivity settings. The measured luminescence was divided by the final OD_600_ to determine the luminescence units per optical density (LU/OD). Each culture was measured in duplicate, and the technical replicates were averaged and plotted as a single biological replicate value. Three independent biological replicates of each set of strains were assayed.

### Surface motility assays

Surface motility assays with strains overexpressing CmrR and CmrT were conducted similarly to prior work (5). Additional details can be found in the supplemental methods.

### AP assays

*C. difficile* strains carrying a *phoZ* phosphatase reporter plasmid were grown overnight in TY-Tm10, then subcultured 1:30 in TY-Tm10 with and without 10 ng/ml ATc for induction. At OD_600_ ~1.0, 2 mL of each culture was pelleted and stored at −20°C. Pellets were thawed on ice, and AP activity was assayed as previously described (6, 36, 37). Additional details can be found in the supplemental methods.

### Purification of CmrR overexpressed in *E. coli*

Expression plasmid pET28(a)+::*cmrR* was transformed into *E. coli* BL21(DE3) for each experiment, and transformants were selected on LB-Kan50 agar. Overnight liquid cultures in LB-Kan50 were used to inoculate 200–500 mL of LB-Kan30 at a 1:30 dilution. Subcultures were grown at 37°C with aeration to an OD_600_ of ~0.5 before IPTG was added to a final concentration of 1 mM. Cultures were shifted to 16°C and grown overnight with aeration. Cells were pelleted by centrifugation and suspended in 50-100 mL of chilled lysis buffer: 1X His6 buffer base (10 mM Tris, 250 mM NaCl, 50 mM NaH_2_PO_4_, 10% glycerol; pH 5.8–6.0 at room temperature) with 1 mM PMSF and 2 mM imidazole. All cells and buffers were kept on ice throughout purification. Cells were lysed by sonication (six cycles of 45 s sonication and 1 minute rest), and lysates were cleared by high-speed centrifugation: 30,000 x *g*, 20 minutes. The supernatant (soluble fraction) was mixed with 1 mL of Ni-NTA resin (Thermo Fisher) and allowed to pass through a 5 mL column (Pierce). The beads were washed with 30 mL of wash buffer consisting of His6 buffer base with 40 mM imidazole. Proteins were eluted in 10 mL of His6 buffer base with 500 mM imidazole. The elution was dialyzed against ~800 mL 1X His6 buffer base three times to remove imidazole, and the protein was stored at 4°C. Samples taken throughout the purification were analyzed by SDS-PAGE and Coomassie staining to ensure successful purification. Protein was quantified by BCA method (Pierce, Thermo Fisher).

### Protein pull-down assays

Strains co-expressing a His-tagged protein from a pET28(a) + vector and a FLAG-tagged protein from a pACYC-FLAG vector were made by co-transforming pairs of plasmids in *E. coli* BL21(DE3) and selecting on LB-Cm20-Kan50 agar. Overnight cultures in LB-Cm10-Kan50 were used to start subcultures in 50 mL LB-Cm10-Kan30 at a 1:30 dilution. For optimal expression, cultures were grown to OD_600_ ~0.5 when expressing one protein (controls) and ~1.0 when expressing two proteins prior to induction with 1 mM IPTG. Cultures were then grown at 16°C overnight with aeration. Cells were pelleted by centrifugation in 50 mL conical tubes (1,000x *g*, 5 minutes, Sorvall Legend RT) and washed with 25 mL phosphate buffered saline (PBS). Pellets were suspended in 25 mL PBS with 1% formaldehyde and incubated on a rocking platform at room temperature for exactly 10 minutes. Fixing was quenched by the addition of 0.5 M glycine for at least 5 minutes, and then cells were washed again with 25 mL PBS. Pellets were stored at −20°C.

Pull-downs were carried out using methods similar to purification of CmrR. In some assays, 1 mM beta-mercaptoethanol was added to all buffers to improve solubility of CmrT. Pellets were suspended in 25 mL of lysis buffer and sonicated. After high-speed centrifugation, the supernatant containing soluble proteins was added to 100 µL of Ni-NTA resin in a 50 mL conical tube. The pellet of insoluble proteins was suspended in 20 mL 1.1X His6 buffer for later analysis. The protein-bead mixture was incubated on a rocking platform for ~2 hours at 4°C to allow binding. Nickel beads were pelleted by brief 1 minute centrifugation at 1,000 x *g*. The supernatant (“flow through,” FT) was carefully removed with a serological pipette, and 5 mL of wash buffer was added to the beads. To wash, the tube was gently swished for ~1 minute before pelleting the beads again. The wash process was repeated for a total of three washes. To elute, 2 mL of elution buffer was added, and the mixture was transferred to a 2 mL microcentrifuge tube. The tube was inverted for 1 minute before pelleting at 1,000 x *g* for 1 minute. The elution was removed carefully by pipetting. Protein samples were mixed 1:1 with 2× SDS sample buffer (125 mM Tris, 20% glycerol, 4% SDS, 0.02 mg/mL bromophenol blue, 10% βME), boiled at 100°C for 10 minutes, and stored at −80°C. Protein samples were separated by electrophoresis on 4%−15% gradient TGX precast gels (Bio-Rad) with Tris-glycine running buffer (25 mM Tris, 192 mM glycine, 0.1% SDS). Identical gels were transferred to 0.45 µM nitrocellulose membranes and analyzed by Western blot. His-tagged proteins were detected with mouse anti-His primary antibody (ABGENT AM1010A) and goat anti-mouse IRDye 680RD secondary antibody (LiCOR 926-68070). FLAG-tagged proteins were detected with mouse anti-FLAG M2 antibody (Sigma F1804) and goat anti-mouse IR800 secondary antibody (Invitrogen SA535521). Western blots were imaged with a LiCOR Odyssey DLx.

### Electrophoretic mobility shift assays

DNA probes were amplified with Phusion polymerase and purified by gel purification (GeneJet Gel Extraction Kit, Thermo Scientific). For EMSAs, 10% acrylamide native gels were poured fresh for each experiment and used within 45 minutes (Protogel, National Diagnostics, 30% (wt/vol) acrylamide). Binding reactions contained: 10 µL of CmrR protein in 1× His6 buffer (various concentrations), 8 µL of DNA probes in water (15 nM final concentration), and 1× Binding Buffer (20 mM Tris pH 8, 1 mM ethylenediaminetetraacetic acid (EDTA), 10 mM MgCl_2_-6H_2_O, 100 mM NaCl, 5% glycerol) added as 2 µL of a 10× stock. Probes amplified from *rpoC* were used as a control for non-specific binding; a short 100 bp probe was included in the same reaction, or a longer 248 bp probe was tested in a separate reaction. Purified CmrR was added at a range of concentrations of approximately 1.5 µM or less for a protein:DNA ratio of ~100:1 or lower. The samples were incubated for 30 minutes at 37°C; simultaneously, the gels were pre-run in a vertical electrophoresis rig (Bio-Rad Mini PROTEAN Tetracell) at 60 V for approximately 30 minutes in 1× native running buffer (192 mM glycine and 25 mM Tris). After the pre-run, the wells of the gels were gently flushed with running buffer. The 20 µL binding reactions mixed with 6 µL of room temperature 4× native sample buffer (250 mM Tris, 40% glycerol, 0.04 mg/mL Bromophenol Blue) were loaded into the wells. A 1kb + DNA ladder (New England Biosciences N3200 or Thermo Scientific SM1343) was loaded for comparison. After electrophoresis for 1 hour with 120 V, gels were stained in one of two ways: GelRed (BIOTIUM) diluted in running buffer and visualized with UV light on a SynGene G-Box Imager (G:Box Chemi XT4) and gel imaging software (GeneSys); or ethidium bromide and visualized with UV light on an Azure 280 imager.

### Bioinformatics

The genome of *C. difficile* R20291 (Genbank Accession FN545816) was searched for the inverted repeat sequence ACAATNNNNNNNNATTGT using MEME Suite’s FIMO program, web version 5.5.5 with default settings (https://meme-suite.org/meme/tools/fimo). Twenty-four exact matches were identified, representing 12 unique sites in the genome. The location of the sites was compared to nearby annotated genes. Six sites were excluded from further analysis because they were not in likely promoter regions, which we defined as being ≤400 bases upstream of an annotated gene’s start codon. The remaining six sites were included in further analysis and are referred to by the number of the nearest downstream gene.

## SUPPLEMENTAL MATERIAL

Supplemental Material jb.00177-25-s0001.pdf Supplemental methods, Tables S1 to S3, and Fig. S1 to S3.

ASM does not own the copyrights to Supplemental Material that may be linked to, or accessed through, an article. The authors have granted ASM a non-exclusive, world-wide license to publish the Supplemental Material files. Please contact the corresponding author directly for reuse.

## References

[1] Guh AY, Mu Y, Winston LG, Johnston H, Olson D, Farley MM, Wilson LE, Holzbauer SM, Phipps EC, Dumyati GK, Beldavs ZG, Kainer MA, Karlsson M, Gerding DN, McDonald LC. 2020. Emerging infections program Clostridioides difficile infection working group. 2020. Trends in U.S. burden of Clostridioides difficile infection and outcomes. N Engl J Med 382:1320–1330. doi:10.1056/NEJMoa1910215 32242357 PMC7861882

[2] CDC. 2019. Antibiotic resistance threats in the United States. Centers for Disease Control and Prevention

[3] Lessa FC, Mu Y, Bamberg WM, Beldavs ZG, Dumyati GK, Dunn JR, Farley MM, Meek JI, Phipps EC, Wilson LE, Winston LG, Cohen JA, Limbago BM, Fridkin SK, Gerding DN. 2015. Burden of Clostridium difficile infection in the United States. N Engl J Med 372:825–834. doi:10.1056/NEJMoa1408913 25714160 PMC10966662

[4] Berumen Alvarez O, Purcell EB. 2023. Expanding our grasp of two-component signaling in Clostridioides difficile J Bacteriol 205:e0018823. doi:10.1128/jb.00188-23 37728603 PMC10601699

[5] Garrett EM, Sekulovic O, Wetzel D, Jones JB, Edwards AN, Vargas-Cuebas G, McBride SM, Tamayo R. 2019. Phase variation of a signal transduction system controls Clostridioides difficile colony morphology, motility, and virulence. PLoS Biol 17:e3000379. doi:10.1371/journal.pbio.3000379 31658249 PMC6837544

[6] Garrett EM, Mehra A, Sekulovic O, Tamayo R. 2021. Multiple regulatory mechanisms control the production of CmrRST, an atypical signal transduction system in Clostridioides difficile. mBio 13:e0296921. doi:10.1128/mbio.02969-21 35164558 PMC8844915

[7] Bourret RB. 2010. Receiver domain structure and function in response regulator proteins. Curr Opin Microbiol 13:142–149. doi:10.1016/j.mib.2010.01.015 20211578 PMC2847656

[8] Gao R, Bouillet S, Stock AM. 2019. Structural basis of response regulator function. Annu Rev Microbiol 73:175–197. doi:10.1146/annurev-micro-020518-115931 31100988

[9] Galperin MY. 2010. Diversity of structure and function of response regulator output domains. Curr Opin Microbiol 13:150–159. doi:10.1016/j.mib.2010.01.005 20226724 PMC3086695

[10] Maule AF, Wright DP, Weiner JJ, Han L, Peterson FC, Volkman BF, Silvaggi NR, Ulijasz AT. 2015. The aspartate-less receiver (ALR) domains: distribution, structure and function. PLoS Pathog 11:e1004795. doi:10.1371/journal.ppat.1004795 25875291 PMC4395418

[11] Al-Bassam MM, Bibb MJ, Bush MJ, Chandra G, Buttner MJ. 2014. Response regulator heterodimer formation controls a key stage in Streptomyces development. PLoS Genet 10:e1004554. doi:10.1371/journal.pgen.1004554 25101778 PMC4125116

[12] Chen X, Alakavuklar MA, Fiebig A, Crosson S. 2023. Cross-regulation in a three-component cell envelope stress signaling system of Brucella. mBio 14:e0238723. doi:10.1128/mbio.02387-23 38032291 PMC10746171

[13] Gao R, Tao Y, Stock AM. 2008. System-level mapping of Escherichia coli response regulator dimerization with FRET hybrids. Mol Microbiol 69:1358–1372. doi:10.1111/j.1365-2958.2008.06355.x 18631241 PMC2586830

[14] Guo XP, Sun YC. 2017. New insights into the non-orthodox two component Rcs phosphorelay system. Front Microbiol 8:2014. doi:10.3389/fmicb.2017.02014 29089936 PMC5651002

[15] Malhotra V, Agrawal R, Duncan TR, Saini DK, Clark-Curtiss JE. 2015. Mycobacterium tuberculosis response regulators, DevR and NarL, interact in vivo and co-regulate gene expression during aerobic nitrate metabolism. J Biol Chem 290:8294–8309. doi:10.1074/jbc.M114.591800 25659431 PMC4375484

[16] Vashist A, Malhotra V, Sharma G, Tyagi JS, Clark-Curtiss JE. 2018. Interplay of PhoP and DevR response regulators defines expression of the dormancy regulon in virulent Mycobacterium tuberculosis J Biol Chem 293:16413–16425. doi:10.1074/jbc.RA118.004331 30181216 PMC6200940

[17] Karimova G, Pidoux J, Ullmann A, Ladant D. 1998. A bacterial two-hybrid system based on a reconstituted signal transduction pathway. Proc Natl Acad Sci USA 95:5752–5756. doi:10.1073/pnas.95.10.5752 9576956 PMC20451

[18] Oliveira Paiva AM, Friggen AH, Qin L, Douwes R, Dame RT, Smits WK. 2019. The bacterial chromatin protein HupA can remodel DNA and associates with the nucleoid in Clostridium difficile. J Mol Biol 431:653–672. doi:10.1016/j.jmb.2019.01.001 30633871

[19] Straughn PB, Vass LR, Yuan C, Kennedy EN, Foster CA, Bourret RB. 2020. Modulation of response regulator CheY reaction kinetics by two variable residues that affect conformation. J Bacteriol 202:e00089-20. doi:10.1128/JB.00089-20 32424010 PMC7348551

[20] Sugadev R, Burley SK, Swaminathan S, New York SGX Research Center for Structural Genomics (NYSGXRC). 2007. Crystal structure of a two-component response regulator from Clostridium difficile. 10.2210/pdb2qzj/pdb

[21] de Been M, Bart MJ, Abee T, Siezen RJ, Francke C. 2008. The identification of response regulator-specific binding sites reveals new roles of two-component systems in Bacillus cereus and closely related low-GC Gram-positives. Environ Microbiol 10:2796–2809. doi:10.1111/j.1462-2920.2008.01700.x 18662309

[22] Bourret RB, Kennedy EN, Tamayo R, Foster CA. 2025. A clarifying perspective on bacterial pseudo-receiver domains. bioRxiv. doi:10.1101/2025.06.23.661182:2025.06.23.661182 PMC12632262 41060066

[23] Desai SK, Kenney LJ. 2017. To ∼P or Not to ∼P? Non-canonical activation by two-component response regulators. Mol Microbiol 103:203–213. doi:10.1111/mmi.13532 27656860 PMC5218973

[24] Fraser JS, Merlie JP Jr, Echols N, Weisfield SR, Mignot T, Wemmer DE, Zusman DR, Alber T. 2007. An atypical receiver domain controls the dynamic polar localization of the Myxococcus xanthus social motility protein FrzS . Mol Microbiol 65:319–332. doi:10.1111/j.1365-2958.2007.05785.x 17573816 PMC1974792

[25] Glanville DG, Han L, Maule AF, Woodacre A, Thanki D, Abdullah IT, Morrissey JA, Clarke TB, Yesilkaya H, Silvaggi NR, Ulijasz AT. 2018. RitR is an archetype for a novel family of redox sensors in the streptococci that has evolved from two-component response regulators and is required for pneumococcal colonization. PLoS Pathog 14:e1007052. doi:10.1371/journal.ppat.1007052 29750817 PMC5965902

[26] Hickey JM, Weldon L, Hefty PS. 2011. The atypical OmpR/PhoB response regulator ChxR from Chlamydia trachomatis forms homodimers in vivo and binds a direct repeat of nucleotide sequences. J Bacteriol 193:389–398. doi:10.1128/JB.00833-10 21057008 PMC3019824

[27] Ruiz D, Salinas P, Lopez-Redondo ML, Cayuela ML, Marina A, Contreras A. 2008. Phosphorylation-independent activation of the atypical response regulator NblR. Microbiology (Reading) 154:3002–3015. doi:10.1099/mic.0.2008/020677-0 18832306

[28] Zannoni A, Pelliciari S, Musiani F, Chiappori F, Roncarati D, Scarlato V. 2021. Definition of the binding architecture to a target promoter of HP1043, the essential master regulator of Helicobacter pylori Int J Mol Sci 22:7848. doi:10.3390/ijms22157848 34360614 PMC8345958

[29] Kaihami GH, Breda LCD, de Almeida JRF, de Oliveira Pereira T, Nicastro GG, Boechat AL, de Almeida SR, Baldini RL. 2017. The atypical response regulator AtvR is a new player in Pseudomonas aeruginosa response to hypoxia and virulence. Infect Immun 85:e00207-17. doi:10.1128/IAI.00207-17 28533471 PMC5520423

[30] Molle V, Buttner MJ. 2000. Different alleles of the response regulator gene bldM arrest Streptomyces coelicolor development at distinct stages. Mol Microbiol 36:1265–1278. doi:10.1046/j.1365-2958.2000.01977.x 10931278

[31] O’Connor TJ, Nodwell JR. 2005. Pivotal roles for the receiver domain in the mechanism of action of the response regulator RamR of Streptomyces coelicolor. J Mol Biol 351:1030–1047. doi:10.1016/j.jmb.2005.06.053 16051268

[32] Tian Y, Fowler K, Findlay K, Tan H, Chater KF. 2007. An unusual response regulator influences sporulation at early and late stages in Streptomyces coelicolor. J Bacteriol 189:2873–2885. doi:10.1128/JB.01615-06 17220225 PMC1855786

[33] McKee RW, Harvest CK, Tamayo R. 2018. Cyclic diguanylate regulates virulence factor genes via multiple riboswitches in Clostridium difficile. mSphere 3:e00423-18. doi:10.1128/mSphere.00423-18 30355665 PMC6200980

[34] McKee RW, Mangalea MR, Purcell EB, Borchardt EK, Tamayo R. 2013. The second messenger cyclic Di-GMP regulates Clostridium difficile toxin production by controlling expression of sigD. J Bacteriol 195:5174–5185. doi:10.1128/JB.00501-13 24039264 PMC3811590

[35] Sekulovic O, Mathias Garrett E, Bourgeois J, Tamayo R, Shen A, Camilli A. 2018. Genome-wide detection of conservative site-specific recombination in bacteria. PLoS Genet 14:e1007332. doi:10.1371/journal.pgen.1007332 29621238 PMC5903667

[36] Anjuwon-Foster BR, Tamayo R. 2017. A genetic switch controls the production of flagella and toxins in Clostridium difficile. PLoS Genet 13:e1006701. doi:10.1371/journal.pgen.1006701 28346491 PMC5386303

[37] Edwards AN, Pascual RA, Childress KO, Nawrocki KL, Woods EC, McBride SM. 2015. An alkaline phosphatase reporter for use in Clostridium difficile. Anaerobe 32:98–104. doi:10.1016/j.anaerobe.2015.01.002 25576237 PMC4385412

