# Supplementary material for: Characterization of two co-regulated response regulators in Clostridioides difficile
Source: J Bacteriol. 2025 Sep 12;207(10):e00177-25. doi: 10.1128/jb.00177-25 (PMC12548384; doi:10.1128/jb.00177-25)
Supplement: Supplemental Material — Supplemental methods, Tables S1 to S3, and Fig. S1 to S3. [file jb.00177-25-s0001.pdf]

Supplemental Materials and Methods

Construction of *C. difficile* overexpression strains

Plasmids with an anhydrotetracycline (ATc)-inducible promoter were used to overexpress *cmrR* or *cmrT* in *C. difficile* as previously described.(1) Point mutations were introduced into pCmrR and pCmrT using primers with changed nucleotides and around-the-world amplification of the entire plasmid with Phusion High-Fidelity DNA Polymerase (Thermo Scientific). All *C. difficile* primers were designed with template sequence of R20291 Genbank accession FN545816. All primers used in this study are listed in Table S2. PCR products were directly transformed into *E. coli* DH5α and plated on Cm20. Single colonies were streaked on selective plates for purity. Purified plasmids were isolated with GeneJet Plasmid Miniprep kit (Thermo Scientific) and confirmed by Sanger sequencing of the modified region. As done previously, plasmids were conjugated into *C. difficile* R20291, R20291 Δ*cmrR,* or R20291 Δ*cmrR*Δ*cmrT* via *E. coli* HB101(pRK24).(2, 3)

Construction of *E. coli* Protein Expression Strains

Codon-optimized versions of *cmrR* and *cmrT* for expression in *E. coli* were purchased from Twist or GenScript. To create constructs with a N-terminal 6xHis tag, codon-optimized versions of *cmrR* and *cmrT* were amplified with Phusion polymerase using primers with added restriction sites. The PCR products were digested with NdeI and BamHI, ligated into similarly digested pET28(a)+ vector, transformed into *E. coli* DH5α, and selected on LB Kan50 agar. pET28(a)+::CDR20291_2188 was purchased from

Twist Biosciences with the codon-optimized gene in the expression vector at the NdeI and XhoI sites. To co-express N-terminally FLAG-tagged proteins in *E. coli*, pACYC-FLAG-dN6-His (gift from Dr. Robert Sauer) was used because the p15A origin and Cm selective marker are compatible with the pBR322 origin and Kan selective marker of pET28(a)+. Expression is controlled by the same IPTG-inducible T7 promoter in both plasmids. Genes were similarly amplified and cloned into pACYC-FLAG using restriction sites NdeI and XhoI. Transformants were selected on Cm20 agar. All plasmids were confirmed by colony PCR (GoTaq Green 2X Master Mix, Promega) and Sanger sequencing.

Construction of *C. difficile* Bacterial Two-Hybrid Assay Strains

We used a bacterial two-hybrid (BacTH) system developed for *C. difficile* that is based on a split luciferase enzyme.(4) Plasmids are constructed with a protein of interest translationally fused to portions of split luciferase named smbit and lgbit. Plasmids with single fusions contain one gene of interested fused to either smbit or lgbit, while the other portion is unfused. Double fusion plasmids contain two gene sequences, one fused to smbit and one to lgbit. Expression of all proteins is controlled by a single ATc-inducible promoter. The *cmrR* and *cmrT* coding sequences fused to the smbit portions of split luciferase were synthesized by IDT DNA Technologies as linear, double stranded fragments (gBlocks). We used Gibson assembly to clone the inserts into SacI and BamHI digested pAP118 plasmid, which has the prior insert removed and serves as an empty vector (Gibson Assembly Master Mix; New England Biolabs, NEB). To create plasmids with lgbit fusions, *cmrR* and *cmrT* sequences were amplified from the smbit

plasmids using primers R2794/2795 and R2794/2796, respectively. The PCR products were digested with PvuI and NotI at the added restriction sites and ligated into similarly digested pAF257 (all enzymes purchased from NEB; T4 DNA Ligase, Invitrogen). Transformants were selected on LB Cm20 agar and confirmed via colony PCR and Sanger sequencing.

Single amino acid mutations were introduced into single fusion plasmids using primers with changed nucleotide(s). Inserts were amplified in two fragments with overlap at the site of the mutation. The internal primers created the mutation and overlapping sequence between the two fragments. Plasmid-specific primers R2899 and R2902 created overlap with the vector backbone. The two fragments were joined by Gibson assembly to create full-length coding sequences, then assembled into SacI and BamHI digested vector. For some plasmids, an “around the world” PCR strategy was used, with the entire plasmid amplified using the primers introducing mutations, and the resulting PCR product directly transformed into *E. coli*.

To create plasmids with two fusion proteins, the smbit and lgbit fusion sequences were taken from the single fusion plasmids by digestion or amplification, and were combined into one plasmid by ligation or Gibson assembly. All plasmids were conjugated into wildtype *C. difficile* R20291 and transformants were selected on BHIS Tm10. Plasmids were confirmed by colony PCR.

Construction of *E. coli* Bacterial Two-Hybrid Assay Strains

An established bacterial two-hybrid system for use in *E. coli* was purchased from Euromedex.(5) To create *cya* fusion constructs, codon-optimized *cmrR* and *cmrT* sequences were excised from pRT2287 and pRT2288, respectively, with KpnI and PstI. Fragments were gel purified, ligated into similarly digested pKNT25 or pUT18, and transformed into DH5α. Transformants were selected on LB Kan100 or Amp100 agar as appropriate, and the plasmids were confirmed by sequencing. To create fusion constructs with alternate alleles of *cmrR* and *cmrT*, inserts were made using splice overlap extension (SOE) PCR. The genes were amplified in two fragments with overlap at the site of the mutation: *cmrR* D52E or *cmrT* E53A. The internal primers created the mutation and overlapping sequence between the two fragments. SOE products were digested with KpnI and PstI, ligated into similarly digested pKNT25 or pUT18, and transformed into DH5α. Transformants were selected on LB Kan100 or Amp100 agar, and the plasmids were confirmed by sequencing. Pairs of plasmids were transformed into *E. coli* BTH101 for use in experiments. Transformants were selected on LB Amp100 Kan50 agar.

Surface motility assays

Surface motility assays with strains overexpressing CmrR and CmrT were conducted similarly to prior work.(1) To measure surface motility, 5 µL of overnight cultures were spotted onto BHIS-1.5% agar. To maintain plasmids, 10 µg/mL Tm was also added, and the indicated concentration of ATc was added to induce expression of plasmid-borne genes. At 3, 5, and 7 days, the size of each spot was determined by calculating the

average of the diameter at the widest part and a perpendicular measurement. Data from three biological replicates conducted simultaneously were analyzed. The plates were photographed using a SynGene G-Box Imager (G:Box Chemi XT4) and imaging software (GeneSys).

Alkaline phosphatase assay

C. difficile strains carrying the *phoZ* phosphatase reporter plasmid were grown overnight in TY Tm10, then subcultured 1:30 in TY with and without 10 ng/mL ATc for induction. At OD_{600} ~1.0, 2 mL of each culture was pelleted and stored at -20°C. Pellets were thawed on ice, and the alkaline phosphatase assay was done as previously described.(6-8) Briefly, pellets were washed with 10 mM Tris, pH 8 and 10 mM $MgSO_4$ and pelleted again. Pellets were suspended in 800 µL assay buffer (1 M Tris HCl, pH 8 and 0.1 mM $ZnCl_2$), 50 µL 0.1% SDS, and 50 µL chloroform. A negative control tube was prepared with the same reagents. All tubes were vortexed for 1 minute, warmed for 5 minutes at 37°C, put on ice for 5 minutes, and pre-warmed to 37°C again. The reaction start time was recorded for each tube upon the addition of 100 µL pNP solution (0.4% *p*-nitrophenyl phosphate in 1 M Tris, pH 8). Tubes were inverted to mix and kept at 37°C until a yellow color was visible. The reaction end time was recorded for each sample upon the addition of 100 µL 1 M KH_2PO_4. Samples were centrifuged for 5 minutes at 13,000 rpm and the OD_{420} and OD_{550} of each sample was measured. Alkaline phosphatase (AP) units were calculated with the equation: $(OD_{420} – (1.75*OD_{550}))*1000) / ((OD_{600}*\text{reaction time})*(\text{volume of cells}))$.

Supplemental Tables and Figures

Table S1. Strains and plasmids used in this study

Lab Notation	Name	Description	Reference
***C. difficile* strains**			
RT273	R20291	Ribotype 027 strain, WT	(9)
RT2256	R20291 Δ*cmrR*	R20291 with in-frame deletion of *cmrR*	(1)
RT2257	R20291 Δ*cmrT*	R20291 with in-frame deletion of *cmrT*	(1)
RT2296	R20291 Δ*cmrR*Δ*cmrT*	R20291 with in-frame deletions of *cmrR* and *cmrT*	(8)
RT2826	R20291 *recV* CDR2492::P*tet*::*cmrR*	R20291 *recV::erm* with ATc-inducible *cmrR* integrated between CDR20291_2492 and CDR20291_2493	(8)
RT2709	R20291 pAF259	Negative control strain for *C. difficile* BacTH, R20291 with pRT2704	This study
RT2710	R20291 pAF262	Positive control strain for *C. difficile* BacTH, R20291 with pRT2705	This study
RT2717	R20291 pCmrR-smbit	R20291 with pRT2712	This study
RT2718	R20291 pCmrT-smbit	R20291 with pRT2713	This study
RT2719	R20291 pCmrR-lgbit	R20291 with pRT2715	This study
RT2720	R20291 pCmrT-lgbit	R20291 with pRT2716	This study
RT2725	R20291 pCmrR-smbit/ CmrR-lgbit	R20291 with pRT2721	This study
RT2726	R20291 pCmrT-smbit/ CmrT-lgbit	R20291 with pRT2722	This study
RT2727	R20291 pCmrR-smbit/ CmrT-lgbit	R20291 with pRT2723	This study
RT2728	R20291 pCmrT-smbit/ CmrR-lgbit	R20291 with pRT2724	This study
RT2732	R20291 pCmrR-D52E-smbit	R20291 with pRT2729	This study
RT2733	R20291 pCmrR-D52A-smbit	R20291 with pRT2730	This study
RT3291	R20291 pCmrR-Y103A-smbit	R20291 with pRT3279	This study
RT3294	R20291 pCmrT-D9A-smbit	R20291 with pRT3282	This study
RT2734	R20291 pCmrT-E53A-smbit	R20291 with pRT2731	This study

RT3297	R20291 pCmrT-T80A-smbit	R20291 with pRT3285	This study
RT3300	R20291 pCmrT-K102A-smbit	R20291 with pRT3288	This study
RT2738	R20291 pCmrR-D52E-lgbit	R20291 with pRT2735	This study
RT2739	R20291 pCmrR-D52A-lgbit	R20291 with pRT2736	This study
RT3292	R20291 pCmrR-Y103A-lgbit	R20291 with pRT3280	This study
RT3295	R20291 pCmrT-D9A-lgbit	R20291 with pRT3283	This study
RT2740	R20291 pCmrT-E53A-lgbit	R20291 with pRT2737	This study
RT3298	R20291 pCmrT-T80A-lgbit	R20291 with pRT3286	This study
RT3301	R20291 pCmrT-K102A-lgbit	R20291 with pRT3289	This study
RT2744	R20291 pCmrR-D52E-smbit/ CmrR-D52E-lgbit	R20291 with pRT2741	This study
RT2745	R20291 pCmrR-D52A-smbit/ CmrR-D52A-lgbit	R20291 with pRT2742	This study
RT3293	R20291 pCmrR-Y103A-smbit/ CmrR-Y103A-lgbit	R20291 with pRT3281	This study
RT3296	R20291 pCmrT-D9A-smbit/ CmrT-D9A-lgbit	R20291 with pRT3284	This study
RT2746	R20291 pCmrT-E53A-smbit/ CmrT-E53A-lgbit	R20291 with pRT2743	This study
RT3299	R20291 pCmrT-T80A-smbit/ CmrT-T80A-lgbit	R20291 with pRT3287	This study
RT3302	R20291 pCmrT-K102A-smbit/ CmrT-K102A-lgbit	R20291 with pRT3290	This study
RT2267	R20291 Δ*cmrR* vector	R20291 Δ*cmrR* with pRT1611	(1)
RT2268	R20291 Δ*cmrR* pCmrR	R20291 Δ*cmrR* with pRT2073	(1)
RT3342	R20291 Δ*cmrR* pCmrR-D52E	R20291 Δ*cmrR* with pRT2074	This study
RT3210	R20291 Δ*cmrR* pCmrR-Y103A	R20291 Δ*cmrR* with pRT3205	This study
RT2307	R20291 Δ*cmrR* Δ*cmrT* vector	R20291 Δ*cmrR* Δ*cmrT* with pRT1611	This study
RT2309	R20291 Δ*cmrR* Δ*cmrT* pCmrT	R20291 Δ*cmrR* Δ*cmrT* with pRT2106	This study

RT3193	R20291 Δ*cmrR* Δ*cmrT* pCmrT-D9A	R20291 Δ*cmrR* Δ*cmrT* with pRT3188	This study
RT2405	R20291 Δ*cmrR* Δ*cmrT* pCmrT-E53A	R20291 Δ*cmrR* Δ*cmrT* with pRT2200	This study
RT3194	R20291 Δ*cmrR* Δ*cmrT* pCmrT-T80A	R20291 Δ*cmrR* Δ*cmrT* with pRT3189	This study
RT3195	R20291 Δ*cmrR* Δ*cmrT* pCmrT-K102A	R20291 Δ*cmrR* Δ*cmrT* with pRT3190	This study
RT3345	R20291 Δ*cmrR* Δ*cmrT* pCmrT-DETK	R20291 Δ*cmrR* Δ*cmrT* with pRT3344	This study
RT2827	R20291 *recV* CDR2492::P*tet*::*cmrR* vector	Inducible *cmrR* strain with pRT1343	(8)
RT2828	R20291 *recV* CDR2492::P*tet*::*cmrR* pMC123::*cmr* TSS4	Inducible *cmrR* strain with pRT2497	(8)
RT3191	R20291 *recV* CDR2492::P*tet*::*cmrR* pMC123::*cmr* TSS4 Δdirect	Inducible *cmrR* strain with pRT3182	This study
RT3192	R20291 *recV* CDR2492::P*tet*::*cmrR* pMC123::*cmr* TSS4 Δinverted	Inducible *cmrR* strain with pRT3183	This study
RT3340	R20291 *recV* CDR2492::P*tet*::*cmrR* pMC123::*cmr* TSS4 Δdirect left	Inducible *cmrR* strain with pRT3338	This study
RT3341	R20291 *recV* CDR2492::P*tet*::*cmrR* pMC123::*cmr* TSS4 Δinverted right	Inducible *cmrR* strain with pRT3339	This study
***E. coli* strains**			
AC472	DH5*α*	F- φ80*lacZ*ΔM15 Δ(*lacZYA*-*argF*)U169 *recA1 endA1 hsdR*17(rκ -, mκ+) *phoA supE44 thi*-1 *gyrA96 relA1* λ- *tonA*	Invitrogen (10)
RT270	HB101(pRK24)	*E. coli* used for conjugation with *C. difficile*, Amp^R	(3)
RT1425	BL21(DE3)	F^- *ompT* $hsdS_B(r_B^-m_B^-)$ *gal dcm*	NEB
RT2272	BTH101	F^- , *cya*-99, *araD139*, *galE15*, *galK16*, *rpsL1* (Str^R), *hsdR2*, *mcrA1*, *mcrB1*	Euromedex
RT2302	BTH101 pKNT25 pUT18	Negative control strain for *E. coli* BacTH –	This study

		BTH101 with empty vectors	
RT2301	BTH101 pKT25-zip pUT18C-zip	Positive control strain for *E. coli* BacTH – BTH101	This study
RT2329	BTH101 pKNT25 pUT18-CmrR	BTH101 with pRT2274 and pRT2294	This study
RT2330	BTH101 pKNT25 pUT18-CmrT	BTH101 with pRT2274 and pRT2295	This study
RT2331	BTH101 pKNT25-CmrR pUT18	BTH101 with pRT2292 and pRT2276	This study
RT2332	BTH101 pKNT25-CmrT pUT18	BTH101 with pRT2293 and pRT2276	This study
RT2365	BTH101 pKNT25 pUT18-CmrR-D52E	BTH101 with pRT2274 and pRT2352	This study
RT2364	BTH101 pKNT25-CmrR-D52E pUT18	BTH101 with pRT2349 and pRT2276	This study
RT2303	BTH101 pKNT25-CmrR pUT18-CmrR	BTH101 with pRT2292 and pRT2287	This study
RT2363	BTH101 pKNT25-CmrR-D52E pUT18-CmrR-D52E	BTH101 with pRT2349 and pRT2352	This study
RT2362	BTH101 pKNT25-CmrR pUT18-CmrR-D52E	BTH101 with pRT2292 and pRT2352	This study
RT2361	BTH101 pKNT25-CmrR-D52E pUT18-CmrR	BTH101 with pRT2349 and pRT2287	This study
RT2306	BTH101 pKNT25-CmrT pUT18-CmrT	BTH101 with pRT2293 and pRT2295	This study
RT2370	BTH101 pKNT25-CmrT-E53A pUT18-CmrT-E53A	BTH101 with pRT2351 and pRT2354	This study
RT2368	BTH101 pKNT25-CmrT pUT18-CmrT-E53A	BTH101 with pRT2293 and pRT2354	This study
RT2369	BTH101 pKNT25-CmrT-E53A pUT18-CmrT	BTH101 with pRT2351 and pRT2295	This study
RT2304	BTH101 pKNT25-CmrR pUT18-CmrT	BTH101 with pRT2292 and pRT2295	This study
RT2305	BTH101 pKNT25-CmrT pUT18-CmrR	BTH101 with pRT2293 and pRT2294	This study
RT2366	BTH101 pKNT25-CmrR-D52E pUT18-CmrT	BTH101 with pRT2349 and pRT2295	This study
RT2367	BTH101 pKNT25-CmrT pUT18-CmrR-D52E	BTH101 with pRT2293 and pRT2352	This study

RT2372	BTH101 pKNT25-CmrR-D52E pUT18-CmrT-E53A	BTH101 with pRT2349 and pRT2354	This study
RT2371	BTH101 pKNT25-CmrT-E53A pUT18-CmrR-D52E	BTH101 with pRT2351 and pRT2352	This study
**Plasmids**			
	pRPF185	*E. coli – C. difficile* shuttle vector with ATc-inducible P_{tet} promoter controlling *gusA*, Cm^R/Tm^R	(11)
pRT2704	pAF259	Split luciferase BacTH positive control plasmid with ATc-inducible full length luciferase (in pRPF185 backbone: *catP*, P_{tet})	(4)
pRT2705	pAF262	Split luciferase BacTH negative control plasmid with split luciferase (P_{tet}-smbit/lgbit)	(4)
pRT2707	pAF257	Split luciferase BacTH negative control plasmid with HupA fused to the lgbit portion of luciferase	(4)
pRT2708	pAP118	Split luciferase BacTH test plasmid with HupA fused to each portion of luciferase	(4)
pRT2712	pCmrR-smbit	Split luciferase BacTH plasmid with CmrR fused to the smbit portion of luciferase	This study
pRT2713	pCmrT-smbit	Split luciferase BacTH plasmid with CmrT fused to the smbit portion of luciferase	This study
pRT2715	pCmrR-lgbit	Split luciferase BacTH plasmid with CmrR fused to the lgbit portion of luciferase	This study
pRT2716	pCmrT-lgbit	Split luciferase BacTH plasmid with CmrT fused to the lgbit portion of luciferase	This study
pRT2721	pCmrR-smbit/ CmrR-lgbit	Split luciferase BacTH plasmid with CmrR fused to each portion of luciferase	This study

pRT2722	pCmrT-smbit/ CmrT-lgbit	Split luciferase BacTH plasmid with CmrT fused to each portion of luciferase	This study
pRT2723	pCmrR-smbit/ CmrT-lgbit	Split luciferase BacTH plasmid with CmrR fused to the smbit portion of luciferase and CmrT fused to the lgbit portion of luciferase	This study
pRT2724	pCmrT-smbit/ CmrR-lgbit	Split luciferase BacTH plasmid with CmrT fused to the smbit portion of luciferase and CmrR fused to the lgbit portion of luciferase	This study
pRT2729	pCmrR-D52E-smbit	Split luciferase BacTH plasmid with CmrR-D52E fused to the smbit portion of luciferase	This study
pRT2730	pCmrR-D52A-smbit	Split luciferase BacTH plasmid with CmrR-D52A fused to the smbit portion of luciferase	This study
pRT3279	pCmrR-Y103A-smbit	Split luciferase BacTH plasmid with CmrR-Y103A fused to the smbit portion of luciferase	This study
pRT3282	pCmrT-D9A-smbit	Split luciferase BacTH plasmid with CmrT-D9A fused to the smbit portion of luciferase	This study
pRT2731	pCmrT-E53A-smbit	Split luciferase BacTH plasmid with CmrT-E53A fused to the smbit portion of luciferase	This study
pRT3285	pCmrT-T80A-smbit	Split luciferase BacTH plasmid with CmrT-T80A fused to the smbit portion of luciferase	This study
pRT3288	pCmrT-K102A-smbit	Split luciferase BacTH plasmid with CmrT-K102A fused to the smbit portion of luciferase	This study
pRT2735	pCmrR-D52E-lgbit	Split luciferase BacTH plasmid with CmrR-D52E fused to the lgbit portion of luciferase	This study

pRT2736	pCmrR-D52A-lgbit	Split luciferase BacTH plasmid with CmrR-D52A fused to the lgbit portion of luciferase	This study
pRT3280	pCmrR-Y103A-lgbit	Split luciferase BacTH plasmid with CmrR-Y103A fused to the lgbit portion of luciferase	This study
pRT3283	pCmrT-D9A-lgbit	Split luciferase BacTH plasmid with CmrT-D9A fused to the lgbit portion of luciferase	This study
pRT2737	pCmrT-E53A-lgbit	Split luciferase BacTH plasmid with CmrT-E53A fused to the lgbit portion of luciferase	This study
pRT3286	pCmrT-T80A-lgbit	Split luciferase BacTH plasmid with CmrT-T80A fused to the lgbit portion of luciferase	This study
pRT3289	pCmrT-K102A-lgbit	Split luciferase BacTH plasmid with CmrT-K102A fused to the lgbit portion of luciferase	This study
pRT2741	pCmrR-D52E-smbit/ CmrR-D52E-lgbit	Split luciferase BacTH plasmid with CmrR-D52E fused to each portion of luciferase	This study
pRT2742	pCmrR-D52A-smbit/ CmrR-D52A-lgbit	Split luciferase BacTH plasmid with CmrR-D52A fused to each portion of luciferase	This study
pRT3281	pCmrR-Y103A-smbit/ CmrR-Y103A-lgbit	Split luciferase BacTH plasmid with CmrR-D52A fused to each portion of luciferase	This study
pRT3284	pCmrT-D9A-smbit/ CmrT-D9A-lgbit	Split luciferase BacTH plasmid with CmrT-D9A fused to each portion of luciferase	This study
pRT2743	pCmrT-E53A-smbit/ CmrT-E53A-lgbit	Split luciferase BacTH plasmid with CmrT-E53A fused to each portion of luciferase	This study
pRT3287	pCmrT-T80A-smbit/ CmrT-T80A-lgbit	Split luciferase BacTH plasmid with CmrT-T80A fused to each portion of luciferase	This study
pRT3290	pCmrT-K102A-smbit/ CmrT-K102A-lgbit	Split luciferase BacTH plasmid with CmrT-	This study

		K102A fused to each portion of luciferase	
pRT2287	pUC57::*cmrR*	Vector with codon optimized *cmrR* sequence for expression in *E. coli*, KanR	This study
pRT2288	pUC57::*cmrT*	Vector with synthesized codon optimized *cmrT* sequence for expression in *E. coli*, KanR	This study
pRT2274	pKNT25	*E. coli* BacTH vector for expression of proteins fused to the T25 fragment of *cya* (adenylate cyclase), KanR	(5)<br>Euromedex
pRT2276	pUT18	*E. coli* BacTH vector for expression of proteins fused to the T18 fragment of *cya* (adenylate cyclase), AmpR	(5)<br>Euromedex
pRT2278	pKT25-zip	*E. coli* BacTH positive control plasmid with leucine zipper domain fused to T25	Euromedex
pRT2279	pUT18C-zip	*E. coli* BacTH positive control plasmid with leucine zipper domain fused to T18	Euromedex
pRT2292	pKNT25-CmrR	*E. coli* BacTH plasmid with codon optimized *cmrR* fused to the T25 portion of adenylyl cyclase	This study
pRT2293	pKNT25-CmrT	*E. coli* BacTH plasmid with codon optimized *cmrT* fused to the T25 portion of adenylyl cyclase	This study
pRT2294	pUT18-CmrR	*E. coli* BacTH plasmid with codon optimized *cmrR* fused to the T18 portion of adenylyl cyclase	This study
pRT2295	pUT18-CmrT	*E. coli* BacTH plasmid with codon optimized *cmrT* fused to the T18 portion of adenylyl cyclase	This study

pRT2349	pKNT25-CmrR-D52E	*E. coli* BacTH plasmid with codon optimized *cmrR*-D52E fused to the T25 portion of adenylyl cyclase	This study
pRT2352	pUT18-CmrR-D52E	*E. coli* BacTH plasmid with codon optimized *cmrR*-D52E fused to the T18 portion of adenylyl cyclase	This study
pRT2351	pKNT25-CmrT-E53A	*E. coli* BacTH plasmid with codon optimized *cmrT*-E53A fused to the T25 portion of adenylyl cyclase	This study
pRT2354	pUT18-CmrT-E53A	*E. coli* BacTH plasmid with codon optimized *cmrT*-E53A fused to the T18 portion of adenylyl cyclase	This study
pRT1611	pRT1611	Derivative of pRPF185 with *gusA* removed, vector control	(6)
pRT2073	pCmrR	pRPF185 with *gusA* replaced with *cmrR*, ATc-inducible expression	(1)
pRT2106	pCmrT	pRPF185 with *gusA* replaced with *cmrT*, ATc-inducible expression	(1)
pRT2074	pCmrR-D52E	pRPF185 with *gusA* replaced with *cmrR*-D52E, ATc-inducible expression	(1)
pRT3205	pCmrR-Y103A	pRPF185 with *gusA* replaced with *cmrR*-Y103A, ATc-inducible expression	This study
pRT3188	pCmrT-D9A	pRPF185 with *gusA* replaced with *cmrT*-D9A, ATc-inducible expression	This study
pRT2200	pCmrT-E53A	pRPF185 with *gusA* replaced with *cmrT*-E53A, ATc-inducible expression	(1)
pRT3189	pCmrT-T80A	pRPF185 with *gusA* replaced with *cmrT*-	This study

		T80A, ATc-inducible expression	
pRT3190	pCmrT-K102A	pRPF185 with *gusA* replaced with *cmrT*-K102A, ATc-inducible expression	This study
pRT3344	pCmrT-DETK	pRPF185 with *gusA* replaced with *cmrT*-D9A-E53A-T80A-K102A, ATc-inducible expression	This study
	pET28(a)+	Expression vector for IPTG-inducible expression, N-terminal His-tag/thrombin/T7-tag and optional C-terminal His-tag, Kan^R	Novagen / EMD Biosciences
pRT2853	pET28(a)+::*cmrR*	pET28(a)+ with codon optimized *cmrR* to express N-terminally His-tagged CmrR in *E. coli*	This study
pRT2861	pET28(a)+::*cmrT*	pET28(a)+ with codon optimized *cmrT* to express N-terminally His-tagged CmrT in *E. coli*	This study
pRT3277	pET28(a)+::2188	pET28(a)+ with codon optimized CDR20291_2188 to express N-terminally His-tagged 2188 in *E. coli,* purchased from Twist Biosciences	This study
pRT3272	pACYC-FLAG-dN6-His	IPTG-inducible expression vector with p15 ori, chloramphenicol resistance marker, and dN6 insert with options for a N-terminal FLAG tag and C-terminal His tag	Addgene #22143, http://n2t.net/addgene:22143; RRID:Addgene_22143 (12)
pRT3273	pACYC::*cmrR*	pACYC-His-dN6-FLAG with dN6 insert replaced with codon optimized *cmrR* to express N-terminally FLAG-tagged CmrR in *E. coli*	This study
pRT3274	pACYC::*cmrT*	pACYC-His-dN6-FLAG with dN6 insert replaced	This study

		with codon optimized *cmrT* to express N-terminally FLAG-tagged CmrT in *E. coli*	
pRT3278	pACYC::2188	pACYC-His-dN6-FLAG with dN6 insert replaced with codon optimized CDR20291_2188 to express N-terminally FLAG-tagged 2188 in *E. coli*	This study
	pMC123	*E. coli – C. difficile* shuttle vector	(3)
pRT1343	pMC123-*phoZ*	pMC123 with *Enterococcus faecalis phoZ*	(6)
pRT2497	pMC123::TSS4-*phoZ*	*phoZ* transcriptional reporter of the region between *cmrR* and right inverted repeat of the *cmr* invertible element	(8)
pRT3182	pMC123::TSS4 Δdirect-*phoZ*	pRT2497 with mutations in both sides of identified direct repeat sequence	This study
pRT3183	pMC123::TSS4 Δinverted-*phoZ*	pRT2497 with mutations in both sides of identified inverted repeat sequence	This study
pRT3338	pMC123::TSS4 Δdirect left-*phoZ*	pRT2497 with mutations in only the left side of identified direct repeat sequence	This study
pRT3339	pMC123::TSS4 Δinverted right-*phoZ*	pRT2497 with mutations in only the right side of identified inverted repeat sequence	This study

Table S2. Primers used in this study

Lab Designation	Name	Use	Sequence[a,b]
***E. coli* Expression**			
R3079	cmrR (CO) pET28a+ fwd	To amplify codon optimized *cmrR* with upstream NdeI	CCCCATATGTACAATTTACTGGTGGTGGATGATGA
R3080	cmrR (CO) pET28a+ rev	To amplify codon optimized *cmrR* with downstream BamHI	CCCGGATCCTTACGGCACAAATTTGTAGCCTTT
R3656	cmrR (CO) pACYC rev	To amplify codon optimized *cmrR* with downstream XhoI	CCCCTCGAGTTACGGCACAAATTTGTAGCCTTT
R3173	cmrT (CO) pET28a+ fwd	To amplify codon optimized *cmrT* with upstream NdeI	AGCCATATGCAGACCAAAATTCTGATTATTGAT
R3174	cmrT (CO) pET28a+ rev	To amplify codon optimized *cmrT* with downstream BamHI	TTCGGATCCTTATTCTTTGTTAAAGTAGTAGCCGG
R3657	cmrT (CO) pACYC rev	To amplify codon optimized *cmrT* with downstream XhoI	CCCCTCGAGTTATTCTTTGTTAAAGTAGTAGCCGG
R3670	pACYC 2188 fwd	To amplify codon optimized 2188 with upstream NdeI	CCCCATATGTATAATATACTAGTAGTTGATGACGATAAGGAAATC
R3671	pACYC 2188 rev	To amplify codon optimized 2188 with downstream XhoI	CCCCTCGAGTTAGAATTTTTCGATTTTATAGCCAACACC
***C. difficile* BacTH**			
R2794	PvuI RBS-GTG	Forward primer to amplify *cmrR* or *cmrT* to make lgbit fusions, includes includes overlap to plasmid for Gibson assembly with upstream PvuI	CCGATCGCTGCAGTAAAGGAGAAAATTTTGTG
R2795	cmrR rev NotI	Adds downstream NotI to amplified *cmrR*	CGCGGCCGCTGGTACAAACTTATATCCTTTTCC
R2796	cmrT rev NotI	Adds downstream NotI to amplified *cmrT*	CGCGGCCGCTTCTTTATTAAAATAATATCCTGCTCCTC
R2899	Split luc up Gib tail	To amplify any gene-smbit fusion	TCGTAGCGTTAACAGATCTGAG
R2900	Smbit rev		GCTATAGAATTTCTTCAAAAAGTCTATAACC
R2901	Smbit fwd	To amplify any gene-lgbit fusion, includes overlap to plasmid for Gibson assembly	GGTTATAGACTTTTTGAAGAAATTCTATAGC
R2902	Split luc dw Gib tail		AAAGTTTTATTAAAACTTATAGGATCCCTAACTG
***E. coli* BacTH**			
R2623	cmrR_co_f1	To amplify codon optimized *cmrR*, upstream PstI site and downstream KpnI site	CACCTGCAGAATGTACAATTTACTGGTG
R2628	cmrR_co_r2		GGTGGTACCTTCGGCACAAATTTGTAG

R2629	cmrT_co_f1	To amplify codon optimized *cmrT*, upstream PstI site and downstream KpnI site	CACCTGCAGAATGCAGAC CAAAATTC
R2632	cmrT_co_r2		GGTGGTACCTTTTCTTTGT TAAAGTAGTAGCC
**CmrR and CmrT mutant alleles**			
R2624	cmrR_co_f2_D 52E	To introduce the D52E mutation into codon optimized *cmrR*	CATCATTTTAGA**A**ATCTCTT TACCGGACATCG
R2626	cmrR_co_r1_D 52E		GGTAAAGAGAT**T**TCTAAAA TGATGCAATCCAG
R2630	cmrT_co_f2_E 53A	To introduce the E53A mutation into codon optimized *cmrT*	ATTTTTCTGG**C**AATTATTCT GACCGACG
R2631	cmrT_co_r1_E 53A		GTCAGAATAATT**G**CCAGAA AAATCAGATCG
RT2529	3128pm_glu_F	To introduce the D52E mutation into *cmrR*	GTATAATTTTGGA**A**ATTTCA TTGCC
RT2530	3128pm_glu_R		GGCAATGAAAT**T**TCCAAAA TTATAC
RT2531	3128pm_ala_F	To introduce the D52A mutation into *cmrR*	GTATAATTTTGG**C**TATTTCA TTGCC
RT2532	3128pm_ala_R		GGCAATGAAATA**G**CCAAAA TTATAC
R3668	CmrR Y103A fwd	To introduce the Y103A mutation into *cmrR*	CAAAACCT**GC**TAGTTTAAA AGAACTTG
R3669	CmrR Y103A rev		CAAGTTCTTTTAAACTA**GC** AGGTTTTG
R3662	CmrT D9A fwd	To introduce the D9A mutation into *cmrT*	GATATTGATAATAG**C**TGGA GACAAGG
R3663	CmrT D9A rev		CCTTGTCTCCA**G**CTATTAT CAATATC
RT2535	3126pm_ala_F	To introduce the E53A mutation into *cmrT*	TTTAG**C**AATAATTCTAACTG ATGGTG
RT2536	3126pm_ala_R		CACCATCAGTTAGAATTAT T**G**CTAAA
R3664	CmrT T80A fwd	To introduce the T80A mutation into *cmrT*	CCAATTGTTTATATG**G**CTTA TATAAATGAAG
R3665	CmrT T80A rev		CTTCATTTATATAAG**C**CATA TAAACAATTGG
R3666	CmrT K102A fwd	To introduce the K102A mutation into *cmrT*	TGATTACTTAATA**GC**GCCT CTAAATTTAG
R3667	CmrT K102A rev		CTAAATTTAGAGGC**GC**TAT TAAGTAATCA
**EMSA**			
R2371	rpoC EMSA_F	Forward primer in rpoC to amplify negative control probes	CATTGTGGTAAATATAGAA GAGTTAG
R3190	rpoC EMSA rev 100 bp	Reverse primer to amplify 100 bp negative control probe	GTCCCATTCTCTCTCTTCTT A

R851	rpoCqR	Reverse primer to amplify 248 bp negative control probe	CCAGTCTCTCCTGGATCAACTA
R2070	OS109	Forward primer probe 2	GGAGATATATGGAGTTAGTGGTGCAA
R2710	cmr5'UTR_R2	Reverse primer probe 2	GCCAAAATTTCACCTATCAATAAAA
R3179	cmr EMSA rev	Reverse primer probes 4, 5, 7, 8	CAACATCATCATCTACAACCAG
R3180	cmr ON EMSA fwd 336 bp	Forward primer probes 5, 6	AAAGGTTTATATTTTGCATCTCAATG
R3181	cmr OFF EMSA fwd 317 bp	Forward primer probes 8, 9	CTAGCCAATAGACAAGTTTCTAG
R3182	EMSA cmr switch up	Forward primer probe 3	GATTTTTATTGATAGGTGAAATTTTGG
R3183	EMSA cmr switch dw	Reverse primer probe 3	ATTGTATTGAACATTAAGATTTATTATTAAATAC
R3184	EMSA cmr TSS4 226 bp	Forward primer probe 7	GTATTTAATAATAAATCTTAATGTTCAATACAAT
R3185	EMSA cmr TSS4 192 bp	Forward primer probe 4	ATTCAAGAATTGTTCAAAAATACTTAAG
R3191	EMSA R3185 reverse	Reverse primer probes 6, 9	CTTAAGTATTTTTGAACAATTCTTGAAT
R3676	EMSA TSS1 upstream	Forward primer probe 1	GATGCTTTAACTTGCAGGGTATTG
R3677	EMSA TSS1 downstream	Reverse primer probe 1	TTGCACCACTAACTCCATATATCTCC
**Promoter Mutations**			
R3680	Mut inv repeat 60 bp fwd	To introduce mutations on both sides of the inverted repeat sequence to create Δinverted	CTTAAGTATTTTTGA**TAGCA**TCTTGAATATT**AC**ATTGAACATTAAGATTTATTATTAAAT
R3681	Mut inv repeat 60 bp rev		ATTTAATAATAAATCTTAATGTTCAAT**GT**AATATTCAAGA**TGCTA**TCAAAAATACTTAAG
R3682	Mut direct repeat 60 bp fwd	To introduce mutations on both sides of the direct repeat sequence to create Δdirect	CTTAAGTATTTTTGAACAATTC**CATCTA**ATTGTA**GATGTTTCA**AAGATTTATTATTAAAT
R3683	Mut direct repeat 60 bp rev		ATTTAATAATAAATCTT**TGAAACATC**TACAAT**TAGATG**GAATTGTTCAAAAATACTTAAG
R3684	Mut direct up fwd	To introduce mutations on one side of the direct repeat	TAATAAATCTT**TGAAACATC**TACAATATTCAAGAATTGTTC

R3685	Mut direct up rev	sequence to create ∆direct left	GAATATTGTA**GATGTTTCA**AAGATTTATTATTAAATACTAATTAAAG
R3686	Mut inv down fwd	To introduce mutations on one side of the inverted repeat sequence to create ∆inverted right	ATACAATATTCAAGA**TGCTA**TCAAAAATACTTAAGTATTATAGTC
R3687	Mut inv down rev		CTTAAGTATTTTTGA**TAGCA**TCTTGAATATTGTATTGAAC

[a] Restriction sites are underlined.

[b] Bases changed to introduce sequence mutations are in red, bolded font.

Table S3. ***E. coli*** **Bacterial two-hybrid strains and results**

	Strain Number	pKNT25 Insert	pUT18 Insert	Result
Controls				
	RT2301	zip	zip	Positive
	RT2302	vector	vector	Negative
	RT2329	vector	CmrR	Negative
	RT2331	CmrR	vector	Negative
	RT2330	vector	CmrT	Negative
	RT2332	CmrT	vector	Negative
	RT2365	vector	CmrR-D52E	Negative
	RT2364	CmrR-D52E	vector	Negative
Homodimers - CmrR				
	RT2303	CmrR	CmrR	Negative
	RT2363	CmrR-D52E	CmrR-D52E	Negative
	RT2362	CmrR	CmrR-D52E	Negative
	RT2361	CmrR-D52E	CmrR	Negative
Homodimers - CmrT				
	RT2306	CmrT	CmrT	Positive
	RT2370	CmrT-E53A	CmrT-E53A	Negative
	RT2368	CmrT	CmrT-E53A	Negative
	RT2369	CmrT-E53A	CmrT	Positive
Heterodimers				
	RT2304	CmrR	CmrT	Negative
	RT2305	CmrT	CmrR	Negative

RT2366	CmrR-D52E	CmrT	Negative
RT2367	CmrT	CmrR-D52E	Negative
RT2372	CmrR-D52E	CmrT-E53A	Negative
RT2371	CmrT-E53A	CmrR-D52E	Negative

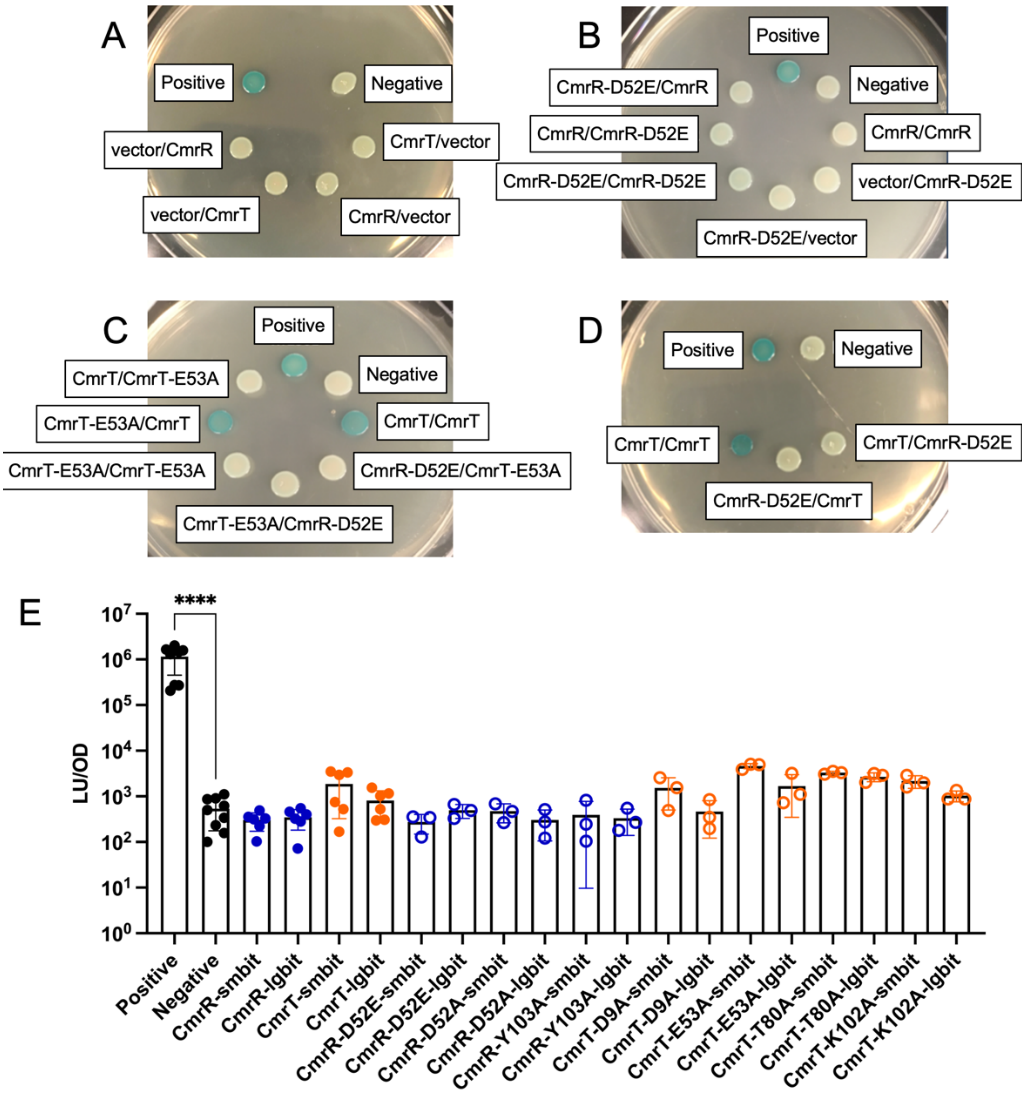

Fig S1. Additional bacterial two-hybrid results. (A-D) A bacterial two-hybrid assay in *E. coli* was used to assess interactions between proteins fused to portions of the adenylate cyclase enzyme encoded by *cya*. Plasmids with T18 and T25 fusion proteins were co-transformed into *E. coli* BTH101. Strains were spotted on LB agar plates with Amp, Kan, IPTG, and X-gal and grown for 48 hours. Representative images are shown with the strains labeled with the relevant protein fusion. All *E. coli* BacTH results are summarized in Table S3. (E) Bacterial two-hybrid in *C. difficile* was used to assess interactions between proteins fused to portions of luciferase enzyme. Strains carrying plasmids with the indicated fusion proteins were grown and luciferase signal was measured. These control strains carried plasmids with a protein of interest fused to only one portion of the split luciferase, i.e., CmrR-smbit is a plasmid with CmrR fused to smbit and nothing fused to lgbit. The positive control strain expresses full length luciferase enzyme, and the negative control strain expresses split luciferase that is not fused to any protein of interest. Data are plotted with mean and SD; $p < 0.0001$, ****; one-way ANOVA compared to negative control with Dunnett’s posttest.

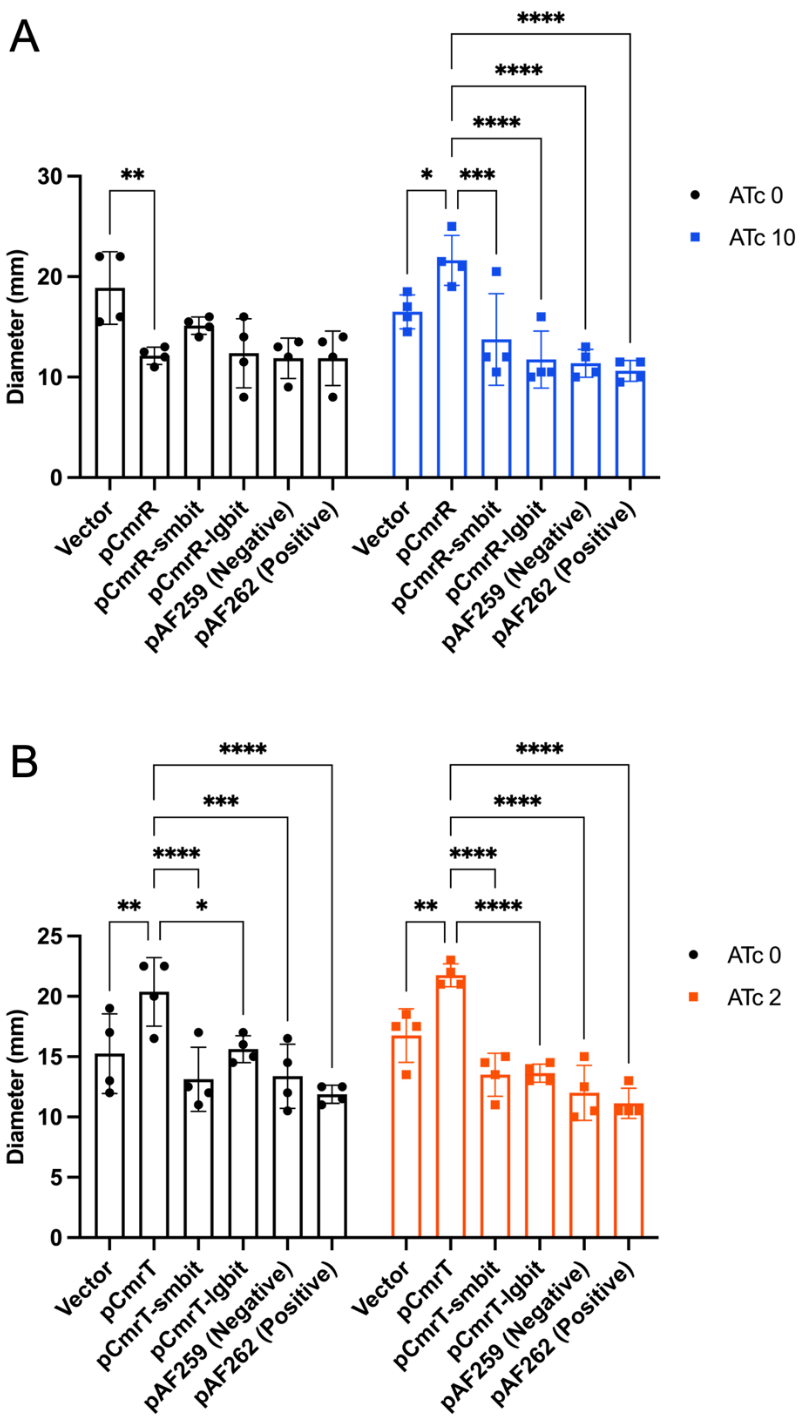

Fig S2. Reduced functionality of CmrR and CmrT with split luciferase tags. Surface motility of strains overexpressing *cmrR* and *cmrT* with protein fusion sequences was assayed by spotting 5 µL of overnight cultures onto BHIS-1.8% agar with 1% glucose, 10 µg/mL Tm, and the indicated concentration of ATc to induce expression of plasmid-borne genes. Data from four biological replicates on day 7 are plotted with the mean and SD. $p < 0.05$, *; $p < 0.01$, **; $p < 0.001$, ***; $p < 0.0001$, ****; two-way ANOVA compared to wildtype allele with Sidak's posttest.

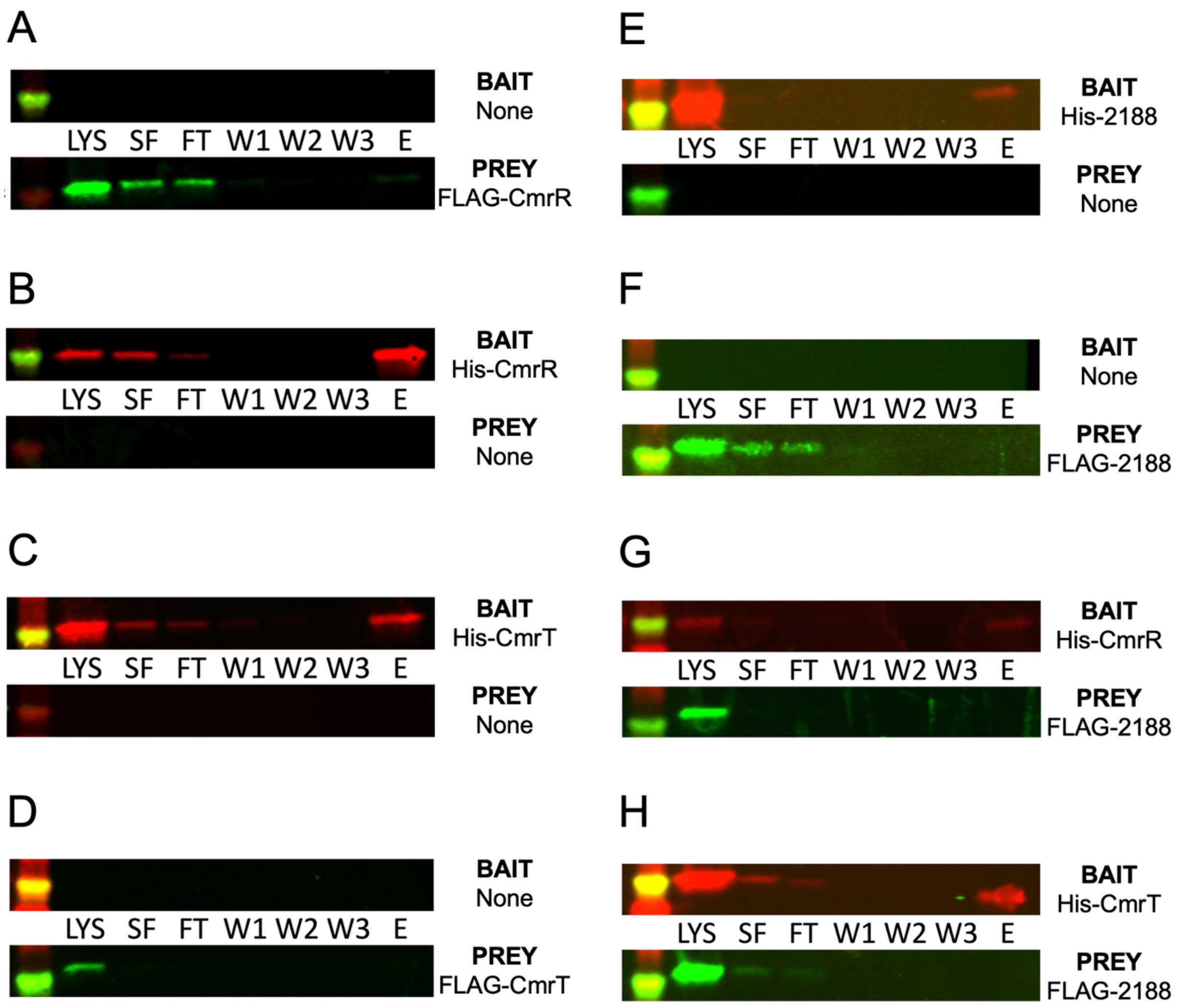

Fig S3. Pull-down negative controls. *E. coli* BL21(DE3) was co-transformed with plasmids carrying 6xHis-tagged bait protein or empty vector and FLAG-tagged prey protein or empty vector (A-F). Binding of CmrR and CmrT to another response regulator, CDR20291_2188, was tested to determine if any two OmpR-family proteins would interact (G-H). Following growth and induction, proteins were purified by nickel affinity. Samples from throughout the purification were loaded on the gel: LYS; soluble fraction, SF; flow through, FT; wash, W; and elution, E. Two identical SDS-PAGE gels were run, transferred to nitrocellulose, and probed with anti-His or anti-FLAG antibodies. The band on the far left is the 25 kDa band of the protein ladder.

References

1. Garrett EM, Sekulovic O, Wetzel D, Jones JB, Edwards AN, Vargas-Cuebas G, McBride SM, Tamayo R. 2019. Phase variation of a signal transduction system controls *Clostridioides difficile* colony morphology, motility, and virulence. PLoS Biol 17:e3000379.
2. Kirk JA, Fagan RP. 2016. Heat shock increases conjugation efficiency in *Clostridium difficile*. Anaerobe 42:1-5.
3. McBride SM, Sonenshein AL. 2011. Identification of a genetic locus responsible for antimicrobial peptide resistance in *Clostridium difficile*. Infect Immun 79:167-76.
4. Oliveira Paiva AM, Friggen AH, Qin L, Douwes R, Dame RT, Smits WK. 2019. The Bacterial Chromatin Protein HupA Can Remodel DNA and Associates with the Nucleoid in *Clostridium difficile*. J Mol Biol 431:653-672.
5. Karimova G, Pidoux J, Ullmann A, Ladant D. 1998. A bacterial two-hybrid system based on a reconstituted signal transduction pathway. Proc Natl Acad Sci U S A 95:5752-6.
6. Anjuwon-Foster BR, Tamayo R. 2017. A genetic switch controls the production of flagella and toxins in *Clostridium difficile*. PLoS Genet 13:e1006701.
7. Edwards AN, Pascual RA, Childress KO, Nawrocki KL, Woods EC, McBride SM. 2015. An alkaline phosphatase reporter for use in *Clostridium difficile*. Anaerobe 32:98-104.
8. Garrett EM, Mehra A, Sekulovic O, Tamayo R. 2021. Multiple Regulatory Mechanisms Control the Production of CmrRST, an Atypical Signal Transduction System in *Clostridioides difficile*. mBio 13:e0296921.
9. Stabler RA, He M, Dawson L, Martin M, Valiente E, Corton C, Lawley TD, Sebaihia M, Quail MA, Rose G, Gerding DN, Gibert M, Popoff MR, Parkhill J, Dougan G, Wren BW. 2009. Comparative genome and phenotypic analysis of *Clostridium difficile* 027 strains provides insight into the evolution of a hypervirulent bacterium. Genome Biol 10:R102.

10. Hanahan D. 1983. Studies on transformation of *Escherichia coli* with plasmids. J Mol Biol 166:557-80.

11. Fagan RP, Fairweather NF. 2011. *Clostridium difficile* has two parallel and essential Sec secretion systems. J Biol Chem 286:27483-93.

12. Martin A, Baker TA, Sauer RT. 2005. Rebuilt AAA + motors reveal operating principles for ATP-fuelled machines. Nature 437:1115-20.
